# Population structure in Neotropical plants: Integrating pollination biology, topography and climatic niches

**DOI:** 10.1111/mec.16403

**Published:** 2022-03-02

**Authors:** Agnes S. Dellinger, Ovidiu Paun, Juliane Baar, Eva M. Temsch, Diana Fernández‐Fernández, Jürg Schönenberger

**Affiliations:** ^1^ 27258 Department of Botany and Biodiversity Research University of Vienna Wien Austria; ^2^ Ecology and Evolutionary Biology University of Colorado Boulder USA; ^3^ QCNE Instituto Nacional de Biodiversidad Quito Ecaudor

**Keywords:** Andes, genetic diversity, melastomataceae, mountain biodiversity, pleistocene climatic fluctuations, pollinator shifts, population differentiation, tropical rain forests

## Abstract

Animal pollinators mediate gene flow among plant populations, but in contrast to well‐studied topographic and (Pleistocene) environmental isolating barriers, their impact on population genetic differentiation remains largely unexplored. Comparing how these multifarious factors drive microevolutionary histories is, however, crucial for better resolving macroevolutionary patterns of plant diversification. Here we combined genomic analyses with landscape genetics and niche modelling across six related Neotropical plant species (424 individuals across 33 localities) differing in pollination strategy to test the hypothesis that highly mobile (vertebrate) pollinators more effectively link isolated localities than less mobile (bee) pollinators. We found consistently higher genetic differentiation (*F*_ST_) among localities of bee‐ than vertebrate‐pollinated species with increasing geographical distance, topographic barriers and historical climatic instability. High admixture among montane populations further suggested relative climatic stability of Neotropical montane forests during the Pleistocene. Overall, our results indicate that pollinators may differentially impact the potential for allopatric speciation, thereby critically influencing diversification histories at macroevolutionary scales.

## 1 INTRODUCTION

Seed plant populations may be linked through two key ecological processes: pollen and seed dispersal (Ballesteros‐Mejia et al., 2016; Cortés et al., 2018; Gelmi‐Candusso et al., 2017; Krauss et al., 2008). Pollen dispersal by animals in particular is considered a major driver of plant population structure, often more important than seed dispersal (Clavino‐Cancela et al., 2012; Gamba & Muchhala, 2020; Kartzinel et al., 2013; Nazareno et al., 2021; Skogen et al., 2019; Yu et al., 2010). Importantly, animal pollinators differ markedly in their mobility, flower‐visitation behaviour and rates of pollen transfer (Bawa, 1992; Breed et al., 2015; Dellinger et al., 2021; Krauss et al., 2017; Levin, 1979). These differences among pollinators may translate into distinct within‐population mating patterns and among‐population differentiation, and may, in theory, differentially affect the probability of subpopulations becoming reproductively isolated and segregated into distinct species (Wessinger, 2021). Despite these possibly far‐reaching effects, we largely lack studies which explicitly evaluate how different pollinators affect population genetic differentiation, particularly so across multiple closely related plant species (and hence considering their shared macroevolutionary history; but see Barbará et al., 2007; Kramer et al., 2011). Such studies are urgently needed not only to better resolve macroevolutionary processes of angiosperm diversification, but also to choose appropriate management strategies in human‐altered, fragmented landscapes and under current climate change (Castilla et al., 2017; Hadley et al., 2012; Toon et al., 2014).

Recently, attempts have been made to formalize theoretical predictions and to provide empirical evidence that pollinator mobility and foraging behaviour significantly impact population genetic structure (Wessinger, 2021). Specifically, less mobile pollinators with small foraging ranges are expected to generate more localized mating patterns between neighbouring individuals (Levin, 1979; Wessinger, 2021). This may result in lower heterozygosity and nucleotide diversity (particularly in small populations, Ness et al., 2010). In self‐compatible species, increased inbreeding may be observed due to repeated visits to the same (geitonogamy) or closely related neighbouring plant individual(s) (Bezemer et al., 2019; Breed et al., 2015; Schoen & Clegg, 1984; Wessinger, 2021). This small‐scale foraging behaviour may lead to strong isolation‐by‐distance even within populations, and particularly high genetic differentiation among populations (Opedal et al., 2016; Schmidt‐Lebuhn et al., 2019; Wessinger, 2021). Small insects, nonflying vertebrates and territorial hummingbirds, with their systematic, repeated visitation of flowers on the same plant(s), fall into this category of “less mobile” pollinators (Medina et al., 2018; Schmidt‐Lebuhn et al., 2019). Highly mobile pollinators, on the other hand, are expected to promote outcrossing and gene flow by visiting dispersed individuals over larger geographical distances, hence resulting in low population differentiation and high admixture among localities (Ballesteros‐Mejia et al., 2016; Gamba & Muchhala, 2020; Hughes et al., 2007; Whelan et al., 2009). Moths and large bees (i.e., carpenter bees) may be considered relatively mobile pollinators since they forage over distances of several hundred metres and more (Castilla et al., 2017; Wikelski et al., 2010). They often adopt a traplining foraging strategy, where they systematically follow a route to visit flowers across spatially dispersed individuals, thereby transferring mixed pollen loads from different donors (Rhodes et al., 2017; Whelan et al., 2009; Wikelski et al., 2010). Traplining is also found in nonterritorial flying vertebrates (e.g., bats, passerines; Fleming, 1982; Tello‐Ramos et al., 2015), and these may be considered as highly mobile pollinators since they often forage over distances of many kilometres and hence may more effectively bridge gaps between isolated populations (Bawa, 1992; Breed et al., 2015; Krauss et al., 2017).

These differences in mobility and foraging strategy of pollinators may further be increased by differences in their susceptibility to abiotic climatic conditions, and their ability to surmount geographical or environmental isolating barriers (Breed et al., 2015; Cruden, 1972; Dellinger et al., 2021). Mountains, in particular, with their highly heterogeneous, rugged terrain, create dispersal barriers and strong abiotic (e.g., geological, climatic) environmental gradients across relatively small spatial and temporal scales (Cortés et al., 2018; Kisel & Barraclough, 2010; Nevado et al., 2018; Surget‐Groba & Kay, 2013). Endothermic vertebrate pollinators may continue foraging across such variable environments since their activity is less affected by, for example, fluctuating, adverse weather conditions common in mountains (Cruden, 1972). Vertebrates may hence pursue their traplining routes across larger areas, and even surmount barriers imposed; that is, by habitat fragmentation or patches of unsuitable habitat (Breed et al., 2015). Ectothermic insect pollinators, on the other hand, are limited in their flower visitation activity to periods of sunny and warm weather (Cruden, 1972), probably further reducing their foraging ranges in mountains.

Besides present‐day pollinator‐mediated gene flow, a species’ current population genetic structure also reflects climatic history and past adaptations to changing abiotic environmental conditions (Cortés et al., 2018; Helmstetter et al., 2020; Ramírez‐Barahona & Eguiarte, 2013; Vasconcellos et al., 2019). Pleistocene glacial cycles, in particular, have led to repeated periods of population isolation, range shifts and habitat recolonization across the globe, leaving imprints in current population genetic diversity and differentiation (Valencia et al., 2010, Escobar et al., 2020; Ornelas et al., 2013; Ortego et al., 2014). These patterns remain poorly understood in the tropics, with idiosyncratic responses documented among different groups of organisms and geographical regions (Ramírez‐Barahona & Eguiarte, 2013; Vasconcellos et al., 2019). Establishing a solid understanding of the geographical and (historical) climatic factors, which may have shaped population structure, is therefore essential when evaluating the relative effect of different pollinators in driving population differentiation across plant species.

**FIGURE 1:**
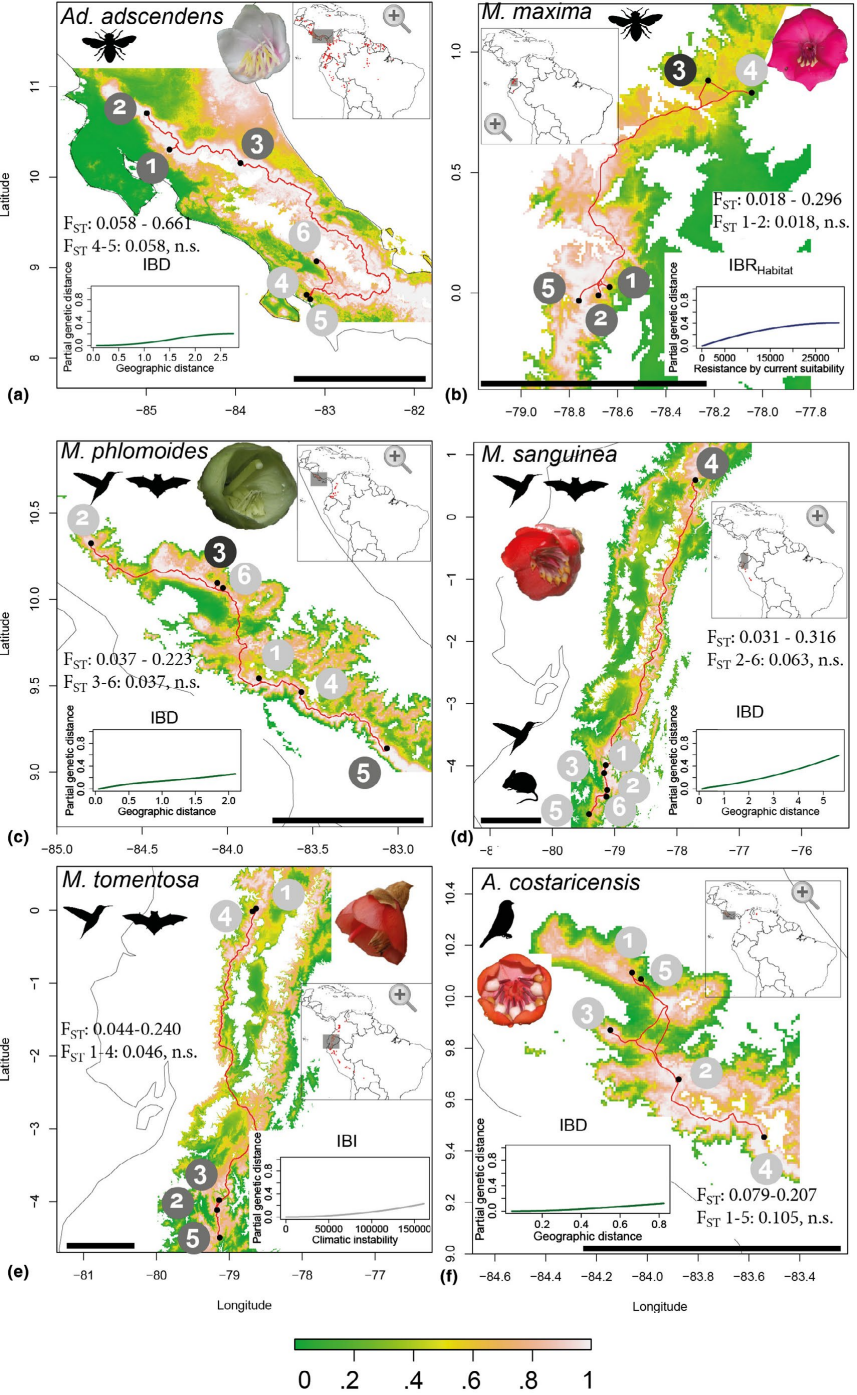
Sampled localities (red dots, grey circles give population ID and clustering as estimated from admixture analyses [Figure 3]) depicted on maps of habitat suitability (IBR_Habitat_, 0—unsuitable, 1—highly suitable). Red lines represent least‐cost distances between study localities, and white areas on maps represent elevations below/above possibly suitable distribution ranges (for habitat suitability across full the distribution, see Figure S3). Per‐species minimum/maximum *F*_ST_ is given, as well as the pairwise *F*_ST_ for the two adjacent populations. I‐spline plots show the variable most strongly explaining population genetic differentiation as estimated through GDM (Table 2). Black bars represent 100 km, black outlines represent coastlines of Costa Rica and Ecuador, and map inserts represent each species’ entire distribution range and the focal study areas. (a) Bee‐pollinated *Adelobotrys adscendens*, lowland rainforests, Costa Rica; (b) bee‐pollinated *Meriania maxima*, montane cloud forests, Ecuador; (c) hummingbird–bat‐pollinated *Meriania phlomoides*, montane cloud forests, Costa Rica; (d) *Meriania sanguinea* with hummingbird–rodent‐pollinated southern populations and hummingbird–bat‐pollinated northern population, cloud forest–Páramo ecotone, Ecuador; (e) hummingbird–bat‐pollinated *Meriania tomentosa*, cloud forests, Ecuador; (f) passerine‐pollinated *Axinaea costaricensis*, cloud forests, Costa Rica

Here, we test the hypothesis that more mobile vertebrate pollinators promote higher levels of outcrossing and less population differentiation than less mobile bee pollinators. Using population genomic approaches based on genotype likelihoods, we contrast six plant species from the Neotropical tribe Merianieae (Melastomataceae). Two of the six species are pollinated by bees, three are pollinated by mixed assemblages of vertebrates (i.e., traplining hummingbirds, bats and rodents) and one is pollinated by nonterritorial passerine birds (Figure 1; Table S1; Dellinger et al., 2014, 2019a, 2021). We aim to understand whether mating patterns are indeed more localized (i.e., lower heterozygosity and nucleotide diversity, higher inbreeding, stronger within‐population isolation‐by‐distance) in localities of bee‐pollinated species than of vertebrate‐pollinated species, and whether flying vertebrate pollinators consistently promote higher levels of gene flow across larger (geographical, topographical, environmental) distances than bees. Given the strong impact of abiotic environmental conditions on the activity of ectothermic bee pollinators, we further expect stronger effects of variation in climatic conditions in bee‐ than in vertebrate‐pollinated species. For each species, we contrast two adjacent populations (<12 km apart) against three to four more distant populations, totalling 424 individuals from 33 localities (Figure 1; Table S1). Since all six species have small, dry, wind‐dispersed seeds, gene flow attributable to seed dispersal should be comparable across the species (and potentially limited to short distances within populations given the dense structure of tropical forests, Kartzinel et al., 2013, Nazareno et al., 2020). Also, all species are self‐compatible, but require pollinator visits to effect pollen release (A. S. Dellinger, unpublished; Dellinger et al., 2019a). Finally, the six species differ in distribution ranges and ecosystems colonized (i.e., lowland rainforests, cloud forests, Figure 1). Our approach of directly addressing these differences through landscape genetics and (historical) niche modelling allows us to objectively evaluate the relative impact of geographical distance, topography, habitat suitability, climatic instability during the Pleistocene and environment on structuring population genetic diversity across a plant group of closely related species with distinct pollination strategies.

## 2 METHODS

### 2.1 Study group

Merianieae (Melastomataceae) are a Neotropical plant tribe of ~300 species, which has radiated recently in the tropical Andes (Dellinger et al., 2021). Bee pollination is ancestral and common both among lowland rainforest and cloud forest Merianieae, while shifts to vertebrate pollination (three times passerine, three times mixed assemblages of vertebrates) are restricted to cloud forest species (for detailed empirical pollinator observations, see Dellinger et al., 2014; 2019b, 2021 and Table S1). For this study, we chose six species: lowland rainforest bee‐pollinated *Adelobotrys adscendens* (Sw.) Triana, cloud forest bee‐pollinated *Meriania maxima* Markgr., passerine‐pollinated *Axinaea costaricensis* Cogn., and three *Meriania* species pollinated by different combinations of vertebrates—*M*. *phlomoides* (Triana) Almeda and *M*. *tomentosa* (Cogn.) Wurdack, pollinated by hummingbirds and bats; *M*. *sanguinea* Wurdack, pollinated by hummingbirds, bats and rodents, Dellinger et al., 2019b. Our sampling covers three independent pollinator shifts from bee to vertebrate pollination (1: *M*. *sanguinea*, 2: *M*. *tomentosa* and *M*. *phlomoides* [part of the same subclade of Merianieae], 3. *Ax*. *costaricensis*; Dellinger et al., 2019b).

### 2.2 Sample collection, GPS coordinates and DNA extraction

We collected leaf and bark material in silica gel and sampled five to six localities per species in Costa Rica (2015) or Ecuador (2016, 2017; Figure 1; Table S1). To ensure comparability between species, we sampled at least two localities in close vicinity (<12 km distance) and the other localities at larger distances (>20 km; Table S2). On average, we sampled 13 (*SD* 3) individuals per locality across a distance of 1 km (Table S1). We recorded the exact location of each individual sampled to assess mating patterns within localities (Gelmi‐Candusso et al., 2017) and calculated the centroid to obtain the average coordinate of each locality. We calculated Euclidean distances between localities based on these averaged coordinates in R (R Core Team, 2020).

We extracted genomic DNA from ~60 mg of dried plant material of 424 individuals using a CTAB protocol with sorbitol washing (Barfuss et al., 2016), RNAse treatment and subsequent clean‐up (gDNA Cleanup Kit; Machery‐Nagl). We quantified double stranded DNA content using a Qubit 3.0 Fluorometer with the dsDNA HS Assay Kit (Thermofisher) and only used samples with more than 6 ng µl^–1^ of DNA.

### 2.3 Genome size estimation and RADseq library preparation

For three species (*Ad*. *adscendens*, *Ax*. *costaricensis*, *M*. *phlomoides*), we estimated genome sizes to select appropriate restriction enzymes for RADseq (restriction site‐associated DNA sequencing). We prepared fresh leaf material for three individuals per species in Otto's buffer (Otto et al., 1981) for propidium iodide flow cytometry (CyFlow ML flow cytometer, Partec; 532‐nm/100‐mW laser, Cobolt Samba, Cobolt AB) following Temsch et al., (2010). We used *Solanum pseudocapsicum* (1C = 1.29 pg DNA) as an internal standard to calculate 1C values for each run (three runs per sample) and calculated the mean 1C per species over all runs and individuals. Average genome sizes (1C value) for the three species were estimated as follows: *Ad*. *adscendens* 0.344 pg, *SD* 0.003; *Ax*. *costaricensis* 0.587 pg, *SD* 0.01; *M*. *phlomoides* 0.639 pg, *SD* 0.006. According to the Kew C‐value database, these genome sizes are the second, third and fourth estimates for the family (Hanson et al., 2001).

In accordance with the relatively small genome sizes, we selected the restriction enzyme *Pst*I (New England Biolabs) for single‐digest RADseq (protocol modified from Paun et al., 2016). We prepared eight RADseq libraries starting with 240 ng DNA per sample, pooling 72 samples per library, including also some repeats (using 120 ng DNA per sample). We ligated 300 nm P1 barcoded adapters (150 nm if 120 ng input DNA) to the restricted samples at 16°C overnight. The P1 adapters included both index and inline barcodes, which were different from each other by at least three nucleotide positions. After P1 ligation, we sheared the DNA by sonication in a Bioruptor Pico (Diagenode) using two cycles (45 s “on,” 30 s“off”; at 4°C) to obtain DNA fragments of 400 bp on average. After P2 adapter ligation, PCR (polymerase chain reaction) amplification and clean‐up steps (using the MiniElute PCR purification Kit, Qiagen), we performed a final size selection for the range 220–850 bp using a 1.5% precasted dye‐free cassette and a Pippin Prep (Sage Science). All libraries were sequenced on an Illumina HiSeq 2500 machine at Vienna BioCenter Core Facilities (VBCF) (http://www.viennabiocenter.org/vbcf/next‐generation‐sequencing/) as 100‐bp single‐end reads.

### 2.4 Identification of RAD loci and variant filtering

We demultiplexed the data to sublibraries (index barcodes) with bamindexdecoder version 1.03 (included in Picard Illumina2Bam, available from http://gq1.github.io/illumina2bam/), and quality‐filtered and further demultiplexed the reads to individual accessions (inline barcodes) using process_radtags.pl from stacks version 1.47 (Catchen et al., 2013). We removed low‐quality reads with an uncalled base and corrected inline barcodes and RAD‐tags with one mismatch, retaining only full‐length reads (94 bp). We concatenated samples which had been sequenced twice. As there is no reference genome available for Merianieae, we followed Brandrud et al., (2020) to first create a pseudoreference using denovo_map.pl from stacks and followed up with a mapping approach to improve coverage and the recovery of loci. For each of the six species, we selected the 10 samples with the highest number of reads to be used for building a unique pseudoreference. We built several catalogues using different settings and chose the best following the r80 rule of Paris et al., (2017) for parameter optimization. We tested *m* (minimum number of identical reads required to create a stack) of 4 and 5, subsequently increased *M* (number of mismatches between stacks within an individual) starting from 1, and *n* (number of mismatches allowed between stacks between individuals) as *n* = *M* or *n* = *M* + 1 (Paris et al., 2017). For each setting, we recorded the number of tags retained with data for at least 80% of individuals and chose the settings *m* = 4, *M* = 1 and *n* = 2 which gave the maximum number of reliable polymorphic loci. We extracted the.fasta pseudoreference from the optimized catalogue by including polymorphic RAD loci that were present in at least 30% of samples and contained up to nine single nucleotide polymorphisms (SNPs) using export_sql.pl from stacks. We then mapped the raw reads of each accession to this pseudoreference separately using the mem algorithm of bwa version 0.7.12‐5 (Li & Durbin, 2009), flagging shorter split hits as secondary (–M). Next, we sorted the resulting aligned.sam‐files by reference coordinates and added read groups in the output.bam‐files with picard tools version 2.18.17 (Wysoker et al., 2013). Finally, we performed a realignment around indels using the Genome Analysis Toolkit version 3.8.1 (gatk; McKenna et al., 2010).

The six species compared in this study are not equally closely related. Specifically, *Ad*. *adscendens*, the lowland bee‐pollinated species, is part of a clade that diverged from core Merianieae (to which the other five species belong) ~25 million years ago (Dellinger, 2021). Whereas the common pseudoreference we used for mapping should be appropriate for the five core Merianieae species, ascertainment bias may affect the inference for *Ad*. *adscendens* based on this reference. To address this issue, we tested whether the mapping rates for the six study species to the pseudoreference differ significantly from each other using ANOVA and Tukey tests as post‐hoc tests. Indeed, the mapping rate of *Ad*. *adscendens* was significantly lower than of all other species (*F* = 86.31, *df* = 5, *p* < .01; for pairwise comparisons see Table S3). We hence decided to create a separate pseudoreference for *Ad*. *adscendens*, based on its own accessions only, following the optimization procedure described above (final settings used are *m* = 5, *M* = 6, *n* = 6). Using this approach, we increased the mapping rate for *Ad*. *adscendens* from 31.7% to 35.1% (Table S4; the reference includes only polymorphic loci). We treated *Ad*. *adscendens* the same way as all other species in subsequent analyses unless otherwise stated.

### 2.5 Mating patterns: Population genetic summary statistics

We measured genetic diversity within localities by calculating per‐site nucleotide diversity (θ_π_, i.e., average number of pairwise differences between sequences) using the genotype‐free method implemented in angsd (Korneliussen et al., 2014). This approach yields more accurate estimates of population genomic parameters from medium to low coverage data like ours (Warmuth & Ellegren, 2019). We generated a theta file for each locality based on the locality‐specific site frequency spectra (SFS). We intersected the theta files of all localities within each species in order to only compare SNPs that were shared by all localities following Peñalba et al., (2018). From these intersected sites files, we calculated nucleotide diversity using thetaStat as implemented in angsd (Maas et al., 2018). We divided θ_π_ estimates by the number of sites and tested for significant differences between localities using Kruskal–Wallis ANOVA and Dunn tests with Bonferroni correction for multiple comparisons (Dinno, 2017). For calculating heterozygosity, we calculated unfolded SFS per individual and divided per‐individual heterozygosity by the total number of sites. Again, we used Kruskal–Wallis ANOVA and Dunn tests to evaluate differences among localities.

We calculated per‐individual inbreeding coefficients (*F*) on genotype likelihoods as the degree of deviation from Hardy–Weinberg equilibrium (ngsf version 1.2.0‐STD, Vieira et al., 2013). We performed two runs in ngsf, the first run to calculate reliable starting values of *F* per individual and a second performing a deeper search, allowing for a maximum of 1,500 iterations.

We assessed localized (within‐locality) mating patterns by testing for isolation‐by‐distance (IBD) between individuals using Mantel's tests on pairwise genetic distances (calculated using ngsdist version 1.0.9, Vieira et al., 2016) and log‐transformed geographical distances between individuals (*mantel*.*randtest*, 10,000 permutations, ade4 version 1.7‐13; Dray & Dufour, 2007).

### 2.6 Population structure: Genetic differentiation and disparity

To visualize population clustering, we used principal component analyses (PCAs), starting from genotype‐frequency‐based covariance matrices (pcangsd version 0.99, Meisner & Albrechtsen, 2018). We further visualized coancestry between individuals for each species using heatmaps (gplots version 3.0.1.1; Warnes et al., 2020).

We used two measures of genetic distance to test whether populations were significantly different from each other. First, we converted pcangsd‐derived covariance matrices to distance matrices (*dist*.*from*.*cov*, rwc version 1.11; Hanks, 2018). Second, we calculated pairwise genetic distances between all individuals of each species (ngsdist). We then tested for significant differences in genetic diversity between populations of the same species (*adonis*, vegan; Oksanen et al., 2019). We used *pairwise*.*adonis* (with corrected *p*‐value estimation) as a post‐hoc test to assess which populations were significantly different. To test whether certain populations were more disparate (genetically variable) than others, we used *betadisper* and TukeyHSD as post‐hoc test (results presented in the Supporting Information).

To assess population genetic differentiation, we calculated *F*_ST_ values on genotype likelihoods starting from unfolded, pairwise SFS (core Merianieae) or folded SFS (*Ad*. *adscendens*) using realSFS in angsd.

We further calculated the admixture coefficient for each individual by estimating the likelihood of genetic clustering in the data (ngsadmix, Skotte et al., 2013). We randomly selected only one SNP per locus and used 10 random initializations to estimate admixture from *K* = 1 to *K* = *n* + 1 ancestral populations, *n* being the total number of sampled localities in each species. We compared the rate of change in the log‐likelihood of different successive *K*‐values (Evanno et al., 2005) to select the *K*‐value best describing clustering in the data and used bar plots for visualization.

### 2.7 Testing for the isolating effects of distance, terrain, current and historical climatic suitability and environmental niche

We used landscape genetic approaches to estimate the degree of connectivity among localities, assessing the relative impact of isolating barriers (IBR_Terrain_, based on topographic complexity), current climatic suitability (IBR_Habitat_, based on environmental niches), climatic suitability since the Last Glacial Maximum (LGM) (IBI, isolation‐by‐instability) and the environmental niche (IBE) on population genomic differentiation (*F*_ST_). For each species’ entire distribution range, we downloaded occurrence data from GBIF (Table S5; Chamberlain et al., 2021, Figure S1) and pruned the data using custom cleaning techniques (coordinatecleaner, Zizka et al., 2019), flagging records with equal longitude/latitude, zero coordinates, coordinate–country mismatches, records located in country centroids, in the sea or around GBIF headquarters, as well as duplicates and altitudinal outliers. To estimate IBR_Terrain_, we used raw elevation data (30 arc‐sec resolution, earthenv.org/topography) and calculated topographic complexity using the Terrain Ruggedness Index (TRI, *tri* function, spatialeco, Evans, 2021). For each species, we drew convex hulls around GBIF occurrences with a 0.4° buffer (rgeos, Bivand & Rundel, 2020) and assessed each species’ elevational distribution range (Table S5). Within the convex hulls, we restricted the TRI layer to the plausible altitudinal distribution range of each species by removing areas 100 m above sea level below and above the lowest and highest GBIF occurrence (*mask*, raster; Table S5). To define a resistance cost surface based on TRI, we chose cost values to represent different proportions of the GBIF occurrences, with 1 (low cost) representing the central 70% of the GBIF occurrences of each species, 2 (moderate cost) representing the adjacent 5% quantiles (10%–15% and 85%–90%), 4 (high cost) representing the next 5% quantiles (5%–10% and 90%–95%), 8 (very high cost) representing the 0%–5% and 95%–100% quantiles, and 16 (extremely high cost) representing TRI values outside of the range covered by actual GBIF occurrences. Next, we calculated a transition object specifying a “knight and one‐cell queen move” direction (*transition*, gdistance, van Etten, 2017) and correcting for map distortion (*geoCorrection*). Finally, we calculated all pairwise least‐cost distances (paths with least‐accumulative cost over cost surface) between the sampling localities (*costDistance* function).

To estimate IBR_Habitat_, we selected four bioclimatic variables which significantly impact pollinator activity and may hence be important in forming environmental barriers (Cruden, 1972; Dellinger et al., 2021): Annual Mean Temperature (bio1), Mean Diurnal Temperature Range (bio2), Annual Precipitation (bio12), and Precipitation Seasonality (bio15; WorldClim 2.0, 30 arc‐sec, Fick & Hijmans, 2017). We restricted these rasters as we did for IBR_Terrain_ and estimated the abiotic climatic niche of each species using all GBIF occurrences using maxent 3.4.1 (dismo, Hijmans et al., 2020). We created 10,000 pseudo‐absence points (the default number of background points used by maxent) from raster cells lacking GBIF occurrences (dismo, Hijmans et al., 2020). We set 80% of the GBIF occurrences of each species as the training data set and 20% to validate the models, using 500 iterations. We fit models 10 times and validated model fit using AUC (area under the receiver operating curve, values >0.75 indicate good fit, Swets, 1988) and TSS (true skill statistic, values range between −1 and +1; −1 to 0 no better than random; values >0.4 to 0.8 acceptable models, see Ornelas et al., 2019, Table S6; sdmtune, Vignali et al., 2020). In addition, we evaluated model performance using fourfold spatial‐block cross‐validation (blockCV, Valavi et al., 2019). In this approach, the occurrence data set is split into four spatially distinct blocks, which are used as training data sets separately and subsequently used to test how well a model can be transferred to environmental space not used for calibration (Table S6). After assuring good model performance, we created raster maps of habitat suitability (0 0% suitable, 1 100% suitable) and averaged all 10 models. To arrive with a resistance cost surface, we subtracted the climatic niche raster from 1 so that 0 means low cost and 1 means high cost. We then calculated the transition object as for IBR_Terrain_ and calculated all pairwise least‐cost distances between the sampled localities.

To estimate IBI, we used the same four bioclimatic variables (bio1, bio2, bio12, bio15, at 2.5 arc‐min resolution) as for the current climate, but derived from three different general circulation models for the LGM and mid‐Holocene (NCAR‐CCSM4, MIROC‐ESM, MPI‐ESM‐P, WorldClim 1.4, Hijmans et al., 2005). Since substantial downward shifts in elevation zones may have occurred during the LGM (Ramírez‐Barahona & Eguiarte, 2013), we set the lower elevation restriction to zero in all species and kept the upper elevation restriction (see above). To minimize errors of temporal extrapolation from current to past niche models, we ran Multivariate Environmental Similarity Surface analyses (MESS) in dismo (Figure S2; Elith et al., 2010). If past environments encompass climatic conditions not found in the training data set, MESS will give negative values. High dissimilarity (many negative values) limits the predictive accuracy of models and hence identifies scenarios/areas where model results need to be treated with particular care. For each species, we calculated MESS for each past circulation model (Figure S2). Dissimilarity was overall low in all species except *M*. *sanguinea*, and the MIROC‐ESM circulation model showed highest dissimilarity across all species (Figure S2). We hence excluded MIROC‐ESM from subsequent analyses.

For estimating habitat stability, we first estimated the current abiotic climatic niche of each species as for IBR_Habitat_, and then projected current habitat suitability to the LGM and mid‐Holocene. We fit each model 10 times and averaged habitat suitability across runs and circulation models. Next, to derive a measure of habitat suitability through time, we overlaid the suitability maps of the three times (current, mid‐Holocene, LGM) and averaged suitability of each raster cell. A raster cell providing 100% suitable conditions at all three times hence received a value of 1, while a raster cell providing unsuitable conditions at all three times received a value of 0. Again, we subtracted the suitability raster by 1 to arrive at a resistance cost surface and calculated all pairwise least‐cost distances between localities of each species (see above; Figure S3).

Finally, we calculated IBE by extracting the raw values of the four bioclimatic variables (current climate) per population and calculating the Euclidean distances in environmental space between sampled localities using the *dist* function in R.

Naturally, the resistance surfaces we calculated for IBR_Terrain_, IBR_Habitat_ and IBI represent refined geographical matrices and may thus be strongly correlated with the Euclidean geographical IBD matrices. We hence first ran separate Mantel tests (with 10,000 permutations) on these four matrices (each matrix standardized by the mean to account for differences in scale) to assess collinearity (García‐Rodríguez et al., 2020). At least two of the four matrices were strongly correlated in all species (Table S7). We hence next constructed separate Mantel tests (with 10,000 permutations) to test for the effect of the respective distance matrix on normalized population genetic differentiation (*F*_ST_/(1 − *F*_ST_)). Then, for each species, we selected the distance measure with the highest *R*² and used multiple matrix regressions with randomizations (10,000 random permutations, Wang, 2013) to test for the relative impact of the respective distance matrix and IBE on population genetic differentiation using *rglMMRR* (popgenreport, Adamack & Gruber, 2014).

Given recent criticism on the use of multiple matrix regressions (inflation of degrees of freedom, weak correlations may receive significant *p*‐values (see discussion on this topic in Moncada et al., 2021), we additionally implemented Generalized Dissimilarity Modelling (GDM). GDM is a multivariate procedure allowing for modelling a single response variable as a function of any number of explanatory variables (Manly, 1986) and allows for nonlinear relationships among the response and explanatory variables using I‐spline functions (Ferrier et al., 2007). For each species, we fit a GDM, specifying *F*_ST_ as the response variable and IBD plus the resistance matrices and IBE as explanatory variables using the R‐package gdm (Fitzpatrick et al., 2021). We plotted I‐splines to visualize how magnitudes and rates of genetic differentiation vary with explanatory variables and estimated the (combination of) explanatory variable(s) best explaining genetic differentiation using backward elimination with permutation (see Ferrier et al., 2007, García‐Rodríguez et al., 2020 for recent implementation). Briefly, the unique contribution of each predictor variable to total explained deviance is calculated, and the variable contributing least is discarded before fitting a new GDM. We ran 500 permutations until all variables retained in the final model made significant (*p* < .05), unique contributions to the explained deviance.

## 3 RESULTS

### 3.1 Genomic data sets

The average number of high‐quality reads retained per accession after demultiplexing ranged between 737,000 and 2,008,000 (Table S4). The final pseudoreferences included 30,791 (*Adelobotrys adscendens*) and 77,720 polymorphic loci (core Merianieae). The average mapping rates ranged between 35.1% and 54.3%, with a coverage of 5.7–16.8× (Table S4). After filtering, we retained 197,868–380,588 variant sites per species (Table S4).

### 3.2 Mating patterns and within‐population IBD

**FIGURE 2:**
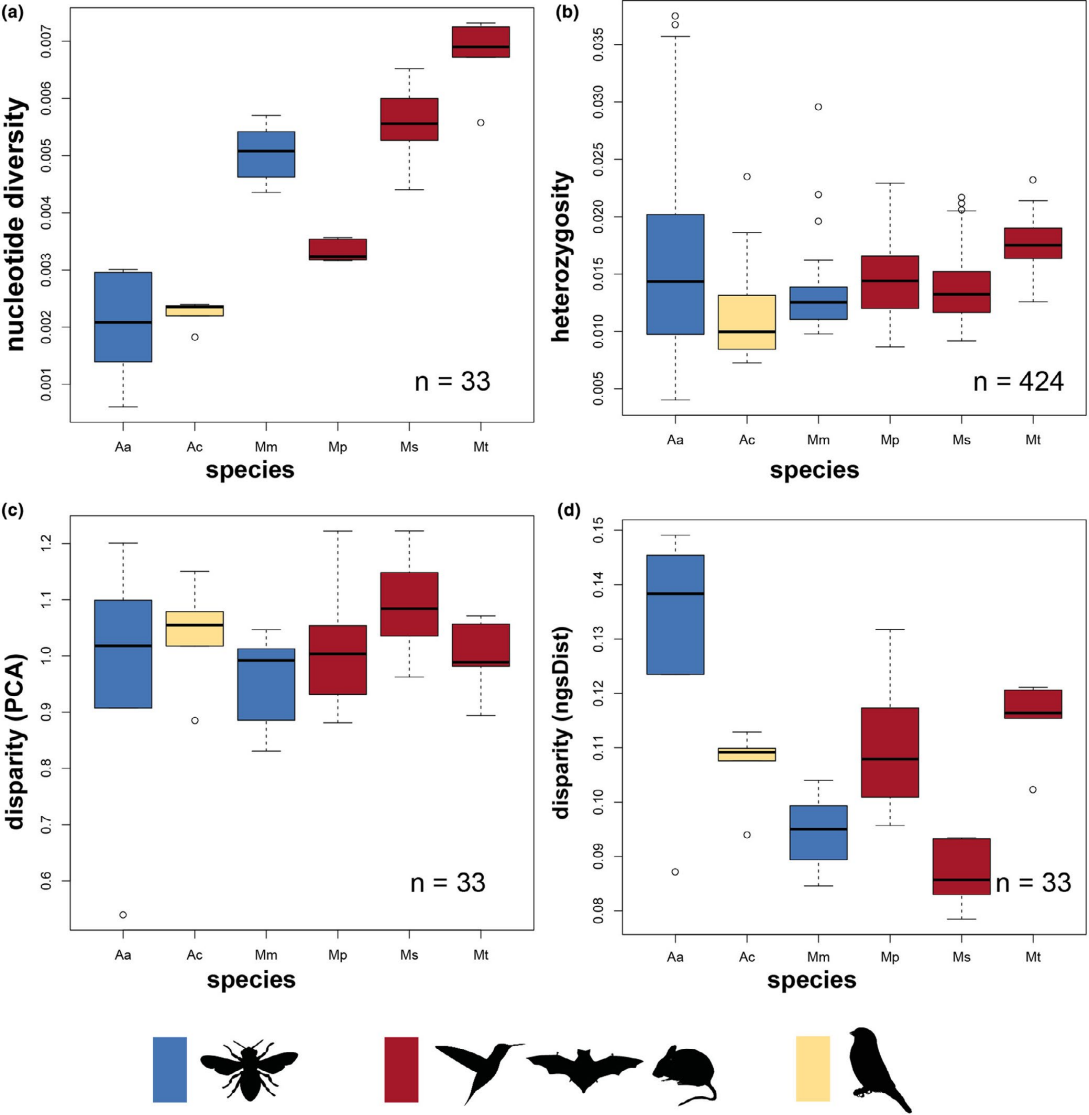
Population genetic summary statistics (per‐site nucleotide diversity, heterozygosity and disparity) for the six study species, colour coded according to pollination strategy. (a) Population nucleotide diversity differed significantly among species, but not among pollination strategies. (b) Individual heterozygosity differed significantly among species, but not among pollination strategies. (c) Disparity (variance in genetic diversity estimated on covariance matrix) did not differ significantly among pollination strategies. (d) Disparity (variance in genetic diversity estimated through ngsdist) did not differ significantly among pollination strategies. Colours indicate the different pollination strategies. *Aa*—*Adelobotrys adscendens*, *Ac*—*Axinaea costaricensis*, *Mm*— *Meriania maxima*, *Mp*— *Meriania phlomoides*, *Ms*— *Meriania sanguinea*, *Mt*— *Meriania tomentosa*

Population nucleotide diversity (θ_π_) across all sites ranged between 0.0006 and 0.0073 and was variable among species (Figure 2a; Tables S8 and S9). Per‐species average estimates were lowest for bee‐pollinated *Ad*. *adscendens* and highest for hummingbird‐bat‐pollinated *Meriania tomentosa* (Table S8). We detected significant differences in θ_π_ both among adjacent and distant localities in all species (Tables S9 and S10).

Heterozygosity was most variable in bee‐pollinated *Ad*. *adscendens* (Figure 2b) and least variable in vertebrate‐pollinated *M*. *tomentosa* (Figure 2b). We detected significant differences in heterozygosity among localities in all species except the vertebrate‐pollinated *Axinaea costaricensis* and *Meriania sanguinea* (Tables S11 and S12). Overall, only few localities differed significantly in heterozygosity (Table S12).

Per‐individual inbreeding coefficients (*F*) were generally low for all species (Figure S4, Table S13). Eleven individuals of *Ad*. *adscendens* showed intermediate to high levels of inbreeding (*F* = 0.05–0.5), followed by *M*. *sanguinea* (one individual) while all other species had lower *F* values (Table S13).

Within localities, there was inconsistent IBD (Table S14). Geographical and genetic distances between individuals correlated significantly in three out of six localities in bee‐pollinated *Ad*. *adscendens*, in two out of five localities in bee‐pollinated *Meriania maxima*, and in one locality each of vertebrate‐pollinated *Meriania phlomoides*, *M*. *tomentosa* and *Ax*. *costaricensis*. There was no significant IBD within localities in *M*. *sanguinea*.

### 3.3 Population structure and disparity

**FIGURE 3:**
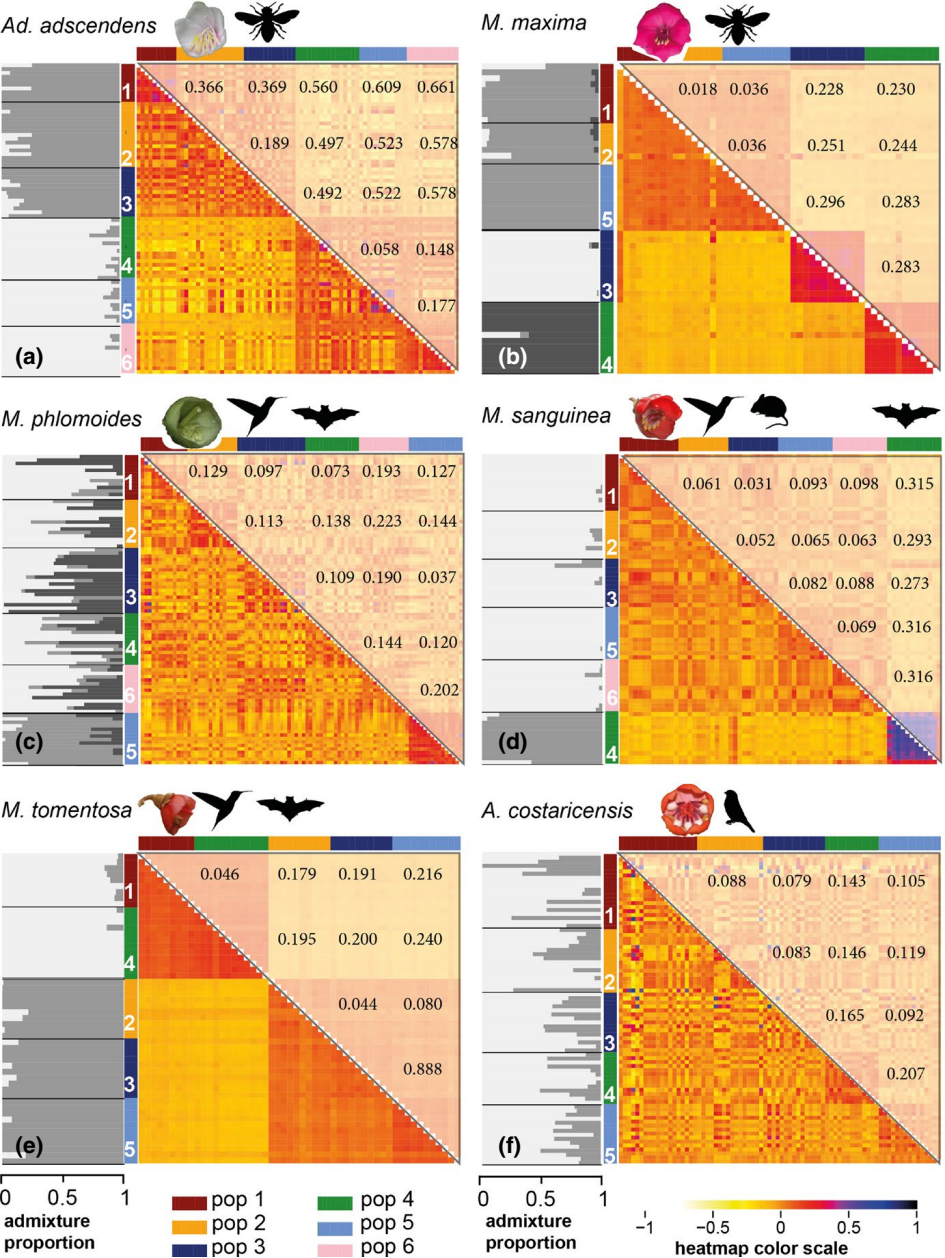
Population structure, co‐ancestry and *F*_ST_ of the six study species. The greyscale bar plots (left) give admixture proportions according to the *K*‐value best describing clustering in the data. In co‐ancestry heatmaps (right, lower triangle), darker tones represent higher pairwise relatedness and stronger differentiation from other individuals; *F*_ST_ values are shown on the upper triangle. Note that (d) and (e) were sampled across much larger distances than the other species. (a) Localities of bee‐pollinated *Adelobotrys adscendens* form two clusters with low admixture proportions; heatmaps indicate relatively high relatedness within clusters, and considerable relatedness among individuals between clusters. (b) Bee‐pollinated *Meriania maxima* with three clusters and very little admixture among clusters. (c) Three ancestral clusters in hummingbird–bat‐pollinated *Meriania phlomoides*, with overall weak clustering, high admixture and high shared co‐ancestry across localities. (d) In hummingbird–bat‐rodent‐pollinated *Meriania sanguinea*, the five hummingbird–rodent‐pollinated localities from southern Ecuador clustered together with low admixture and shared co‐ancestry across localities. They were significantly different from locality 4 (northern Ecuador, hummingbird–bat‐pollinated, Table S15) and also showed floral adaptations to their different nocturnal (rodent/bat) pollinators (Table S1; Dellinger et al., 2019b). (e) Two ancestral clusters with low admixture in hummingbird–bat‐pollinated Meriania tomentosa, with low shared co‐ancestry between those clusters. (f) All localities were intermixed without clear clustering (best *K* = 2) in passerine‐pollinated *Axinaea costaricensis*, with considerable admixture and shared co‐ancestry among all localities

Population structuring was variable between species and clustering mostly reflected geographical relationships (Figure 3; Figure S5). Individuals of lowland bee‐pollinated *Ad*. *adscendens* grouped into two distinct clusters (South‐Western versus Northern Costa Rica, Figures 1a and 3a). While admixture analyses supported two ancestral populations with low admixture, co‐ancestry estimates revealed shared co‐ancestry between the two geographical clusters (Figure 3a). In bee‐pollinated *M*. *maxima*, individuals clustered into three distinct groups (Figure 3b) despite relative geographical vicinity among all localities (Figure 1). Admixture analyses detected three ancestral populations with low admixture (<10%) and were supported by co‐ancestry estimates (Figure 3b). We also detected population clustering in hummingbird–bat–rodent‐pollinated *M*. *sanguinea* (two clusters, Figure 3d) and in hummingbird–bat‐pollinated *M*. *tomentosa* (three clusters, Figure 3e), representing distant localities in northern and southern Ecuador (Figure 1). Admixture was mostly below 20% in both species, with highest probability of two ancestral populations, and low co‐ancestry between the geographical clusters (Figure 3d,e). Population structuring was weak and admixture high (~30%) in hummingbird–bat‐pollinated *M*. *phlomoides* (Figure 3c) and passerine‐pollinated *Ax*. *costaricensis* (Figure 3f). We found highest likelihood for three ancestral populations in *M*. *phlomoides*, and two ancestral populations in *Ax*. *costaricensis*, and overall high shared co‐ancestry across localities (Figure 3c,f; Figure S5).

In all species, at least some localities were significantly differentiated from each other (Tables S15 and S16), but adjacent localities were generally not differentiated (Figure 1). Genetic differentiation was weakest in passerine‐pollinated *Ax*. *costaricensis* and highest in bee‐pollinated *Ad*. *adscendens*. We detected most significant differences in genetic disparity among localities in *Ad*. *adscendens* (6/15 comparisons) and *M*. *tomentosa* (4–5/10 comparisons) and no differences in *M*. *sanguinea* and *Ax*. *costaricensis* (Tables S17 and S18). Compared to the other species, montane bee‐pollinated *M*. *maxima* showed lowest disparity within localities (Figure S5).

### 3.4 IBD, resistance, instability and environment

Pairwise population fixation indices (*F*_ST_) confirmed patterns found in admixture and co‐ancestry analyses and indicated moderate to high population structuring, ranging from 0.0178 (adjacent localities 1 and 2 of *M*. *maxima*) to 0.661 (distant localities 1 and 6 of *Ad*. *adscendens*; Figure 1; Table S2). On average, across all localities in each species, *Ax*. *costaricensis* showed lowest (*F*_ST_ = 0.123) and *Ad*. *adscendens* highest genetic differentiation (*F*_ST_ = 0.422). Comparing the two adjacent localities in each species, we found lowest *F*_ST_ in bee‐pollinated *M*. *maxima* (0.018) and highest in passerine‐pollinated *Ax*. *costaricensis* (0.105, Figure 1).

**FIGURE 4:**
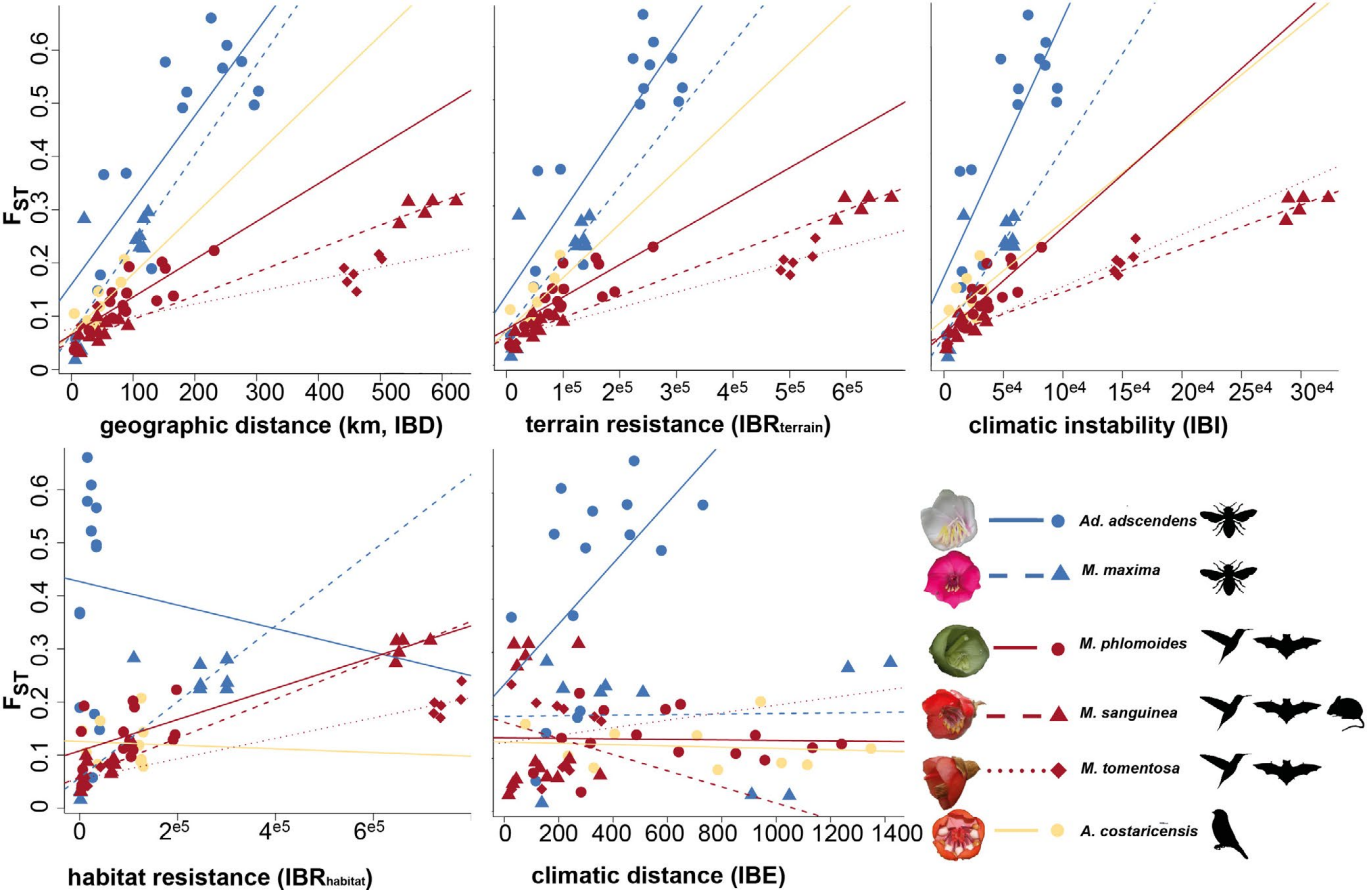
Relationship between genetic distance (*F*_ST_) and geographical distance (IBD), topographic barriers (IBR_Terrain_), climatic instability (IBI), habitat suitability (IBR_Habitat_) and climatic distance (IBE) in the six study species. We detected significant IBD/IBR/IBI in all species except *Axinaea costaricensis*, and no IBE in any species. Our results suggest that large geographical distances among localities of species pollinated by less mobile bee pollinators (blue) result in larger genetic differentiation than in species pollinated by mixed assemblages of (volant) vertebrates (red, yellow). Correlations of genetic distance and current habitat suitability and climatic variables were (mostly) not significant. Relationships are depicted for each species separately; each dot represents a pairwise population comparison

Mantel's tests on normalized *F*_ST_ revealed that, in all species, population differentiation was strongly associated with geographical distance (IBD, Table 1, Figure 4). Associations were significant in all species except *Ax*. *costaricensis* (*R*² = .75, *p* = .055). In vertebrate‐pollinated *M*. *phlomoides*, *M*. *sanguinea*, *M*. *tomentosa* and *Ax*. *costaricensis*, *F*_ST_ also correlated most strongly with geographical distance (IBD). In bee‐pollinated *Ad*. *adscendens*, correlation was strongest (but not significant) with geographical barriers (IBR_Terrain_) and historical habitat suitability (IBI, *R*² = .796, *p* = .048), while in *M*. *maxima*, *F*_ST_ was associated most strongly with current climatic suitability (*R*² = .777, *p* = .023, IBR_Habitat_). In all species except *Ax*. *costaricensis*, we also detected a significant correlation with Pleistocene climatic instability (IBI, Table 1; Figures S3, S6 and S7). We did not detect any significant correlation with environmental distances (IBE, Figure 4, Table 1). These patterns were mostly confirmed by multiple matrix regressions, although effects of IBI/ IBR_Habitat_ were not significant in *Ad*. *adscendens* and *M*. *maxima*, respectively (Table S19).

**TABLE 1:** Results of Mantel's tests on the impact of IBD, IBR_Terrain_, IBR_Habitat_, IBI and IBE on normalized population genetic differentiation (*F*_ST_). Significant isolation by distance and/or resistance was observed in all species except *Axinaea costaricensis*, no IBE, the highest significant *R*² for each species is highlighted in bold, and significant values are given in italics

Species	IBD		IBRTerrain		IBRHabitat		IBI		IBE	
	*R*²	*p*	*R*²	*p*	*R*²	*p*	*R*²	*p*	*R*²	*p*
*Adelobotrys adscendens*	. ***785***	. ***047***	.821	.053	.017	.470	.*796*	.*048*	.556	.055
*Meriania maxima*	.*687*	.*017*	.*673*	.*017*	. ***777***	. ***023***	.*729*	.*016*	.091	.394
*Meriania phlomoides*	. ***815***	. ***006***	.*784*	.*008*	.363	.171	.*808*	.*002*	−.108	.622
*Meriania sanguinea*	. ***987***	. ***005***	.*987*	.*007*	.*984*	.*01*	.*985*	.*011*	−.150	.348
*Meriania tomentosa*	.**951**	.**015**	.*948*	.*016*	.*941*	.*024*	.*950*	.*017*	−.016	.434
*Axinaea costaricensis*	.**750**	.**055**	.675	.074	−.002	.547	.417	.073	−.093	.632

These results were mostly confirmed by GDM (Table 2; Tables S20 and S21 detail model fit). In bee‐pollinated *Ad*. *adscendens*, we detected strong effects of IBD, IBR_Terrain_ and IBE on *F*_ST_ (Figure S8), although only IBD was retained as significant in the final model (Table 2). In bee‐pollinated *M*. *maxima*, we found significant effects of current habitat suitability (IBR_Habitat_) and IBD (Table 2; Figure S9). *F*_ST_ in hummingbird–bat–rodent‐pollinated *M*. *sanguinea* and hummingbird–bat‐pollinated *M*. *phlomoides* was significantly affected only by geographical distance (IBD, Table 2; Figures S10 and S11), while *F*_ST_ in hummingbird–bat‐pollinated *M*. *tomentosa* was most strongly affected by climatic instability since the LGM (IBI) and IBD (Table 2; Figure S12). In *Ax*. *costaricensis*, again, no variable significantly affected *F*_ST_ (Table 2; Figure S13).

**TABLE 2:** Importance (Imp.) of IBD, IBR_Terrain_, IBR_Habitat_, IBI and IBE in explaining population genetic differentiation (*F*_ST_) as assessed through GDM (compare Table S20, Figures S4–S10); Imp.‐1/‐2/‐3 give models where, sequentially, the respective explanatory variable(s) contributing least to model fit were removed (indicated by empty cell). Note that models may fail to fit if relationships between *F*_ST_ and (some) explanatory variables are weak (indicated by “/”)

	Imp.	*p*	Imp.−1	*p*	Imp.−2	*p*	Imp.−3	*p*
***Adelobotrys adscendens***								
IBD	*0.208*	.*016*	*0.208*	.*005*	*0.723*	.*014*	*64.328*	.*014*
IBRTerrain	0.237	.146	0.237	.150	0.238	.162		
IBRHabitat	0	.814						
IBI	0.000	.346	0.000	.324				
IBE	0.817	.172	0.817	.156	0.817	.158	2.710	.184
***Meriania maxima***								
IBD	*0.022*	*<.001*	*0.022*	*<.001*	*0.022*	*<.001*	*0.274*	*<.001*
IBRTerrain	0	1						
IBRHabitat	10.869	.226	10.869	.230	*30.853*	*<.001*	*30.637*	*<.001*
IBI	0	1	0	1				
IBE	0.311	.614	0.311	.628	0.311	.600		
***Meriania phlomoides***								
IBD	*2.393*	.*008*	*2.777*	.*008*	*2.777*	.*005*	*99.919*	.*016*
IBRTerrain	0	1						
IBRHabitat	0	1	0	1				
IBI	0.005	.512	0.005	.496	0.005	.506		
IBE	0.398	.370	0.398	.316	0.398	.215	/	/
***Meriania sanguinea***								
IBD	*0.035*	.*021*	*0.035*	.*016*	*0.687*	.*016*	/	/
IBRTerrain	0	.830	0	.836				
IBRHabitat	0.000	.514	0.000	.496	0.000	.490		
IBI	0.000	.832						
IBE	0.861	.134	0.861	.168	0.879	.200	0.879	.146
***Meriania tomentosa***								
IBD	*2.193*	*<.001*	*2.193*	*<.001*	*2.193*	*<.001*	*2.193*	*<.001*
IBRTerrain	0	1						
IBRHabitat	0	1	0.000	1	0.000	1		
IBI	0.225	.410	1.157	.206	1.157	.170	*55.832*	*<.001*
IBE	0	1	0	1				
***Axinaea costaricensis***								
IBD	14.532	.309	68.258	.310	68.258	.334	/	/
IBRTerrain	0	1						
IBRHabitat	0	1	0	1				
IBI	0	1	0	1	0	1		
IBE	0	1	0	1	0	1	0	1

## 4 DISCUSSION

Here we tested the hypothesis that pollination by less mobile insect pollinators results in higher population differentiation and more localized mating patterns than pollination by highly mobile flying vertebrates. We explore this hypothesis across a sample of six closely related Neotropical plant species with disparate pollination systems. Assessing geographical, climatic and topographic isolating barriers, we detected consistently stronger isolating effects (particularly IBD) across localities of bee‐ than vertebrate‐pollinated species (Table 1, Figure 4). These results support the hypothesis that more mobile pollinators (i.e., flying vertebrates) may connect populations more effectively than less mobile (i.e., [small] bee) pollinators (Medina et al., 2018; Wessinger, 2021). Even though large bees (pollinators of *Meriania maxima*) are generally considered as relatively mobile (Gamba & Muchhala, 2020; Wikelski et al., 2010), their flight activity is strongly reduced under adverse weather conditions in tropical mountains, probably limiting (large‐distance) pollen dispersal (Dellinger et al., 2021). Within localities, we also detected IBD more frequently among bee‐ than vertebrate‐pollinated individuals (Table S14). In contrast to the idea that less mobile pollinators would promote more localized mating patterns, we did not find consistent differences in nucleotide diversity, heterozygosity or disparity according to pollination strategy (Figure 2). This suggests that, at least across small spatial scales (i.e., within localities, between adjacent localities), bees may be equally effective outcrossing pollinators as vertebrates (Castilla et al., 2020; Opedal et al., 2016, Schmidt‐Lebuhn et al., 2019). Clearly, within‐population genetic diversity may also be affected by factors other than pollinator foraging behaviour. The overall size of a population, for example, is generally positively correlated with genetic diversity (Hague & Routman, 2015). Furthermore, differences in wind properties (i.e., wind speed, direction) among localities of the same species may impact the dispersal of fruits or seeds (such as, for instance, the tiny, wind‐dispersed seeds of Merianieae), leading to pollen‐dispersal‐independent, spatial patterns of genetic diversity and clustering of individuals within populations (Mueller‐Landau et al., 2008). We also want to emphasize that the set‐up of our study with only two bee‐ vs. four vertebrate‐pollinated species from two different biogeographical regions may limit the explanatory power of our results. While this set‐up allowed us to also address alternative, pollinator‐independent factors potentially influencing the present‐day population genetic structure of our study group, it is clear that additional studies based on larger sample sizes of bee‐ and vertebrate‐pollinated species from other tropical plant clades are needed to evaluate the generality of the patterns reported here.

Our study species differ in distribution ranges and ecosystems colonized. Bee‐pollinated *Adelobotrys adscendens* has a wide distribution in lowland rainforests in tropical Latin America, while the other five species inhabit montane cloud forests with relatively continuous (*Meriania phlomoides*, *M*. *tomentosa*) or patchy (*Axinaea costaricensis*, *M*. *maxima*, *M*. *sanguinea*) distributions (Figure S1). We may, hence, expect partly idiosyncratic responses to isolating barriers and (current and past) climatic habitat suitability. Following the expectation that mountain terrain generates strong physical barriers (Barbará et al., 2007), we did indeed detect significant IBR_Terrain_ in all species except *Ax*. *costaricensis* (Table 1). It was, however, never recovered as a factor best explaining population differentiation (Table 2). While rugged mountain terrain may act as effective barrier even across small spatial scales (i.e., populations 3 and 6 in *M*. *phlomoides*, Figure 1), admixture was overall high among close (<12 km) localities in all species, suggesting considerable connectivity by both insect and vertebrate pollinators among localities (Figure 3; Castilla et al., 2017; Tello‐Ramos et al., 2015).

Ectothermic insect pollinators may be affected more strongly by harsh abiotic climatic conditions (i.e., low temperatures, high precipitation, strong winds) than vertebrate pollinators (Cruden, 1972; Dellinger et al., 2021). Accordingly, we found some isolating effects related to current climatic conditions (Table 2, Figure 4) only in the two bee‐pollinated species (weak IBE in *Ad*. *adscendens* and IBR_Habitat_ in *M*. *maxima*). In *Ad*. *adscendens*, the marked separation into two clusters (Figures 1a and 3a) clearly reflects the combination of IBD, isolating effects related to mountain topography and current climatic conditions. The southwestern localities are cut off from the northeastern localities by the central Costa Rican mountain range. These mountains feature moist and cool cloud forests, which are generally unhostile to smaller bees, the pollinators of *Ad*. *adscendens* (Dellinger et al., 2019c). Along the western coast, occasional dry habitats (i.e., Nicoya peninsula), on the other hand, represent unsuitable habitats for the moisture‐adapted plants (Pröhl et al., 2010). In accordance with this, our niche models estimated the “least‐cost” path connecting the southern and northern localities of *Ad*. *adscendens* through the southeastern lowlands along the Caribbean side of the high Central American mountains (Figure 1a; Patten & Smith‐Patten, 2008). In *M*. *maxima*, the marked differentiation among localities 3 and 4 further correlates with environmental differences. These two localities were significantly differentiated from each other genetically, albeit only 20 km apart. Our habitat‐suitability models indicated that locality 4 lies in a climatically less suitable area (Figure 1). It is therefore possible that a difference in habitat suitability strongly reduced the probability of bee flight among these localities (given reduced bee activity under adverse montane weather conditions, Dellinger et al., 2021), hence generating strong population differentiation. Interestingly, we also found individuals of *M*. *maxima* in locality 4 to differ morphologically (i.e., smaller flowers, nonrevolute leaf basis; A. S. Dellinger, pers. observ.). Whether these observed phenotypic differences are the result of random genetic drift, or a response to different selection pressures, remains to be investigated.

Among the vertebrate‐pollinated species, we did not find isolating effects caused by habitat suitability (IBR_Habitat_) or environment (IBE; Figure 1, Table 2). Indeed, cloud forests form a relatively continuous ecosystem, particularly on the eastern slopes of the Andes and Central American mountains (Balslev, 1988; Luebert & Weigend, 2014), and possibly provide continuously suitable habitats for the cold‐adapted vertebrate pollinators. The marked population differentiation observed between northern and southern Ecuadorian populations of *M*. *sanguinea* and *M*. *tomentosa*, on the other hand, follows the well‐known biogeographical barrier of the dry Girón–Paute valley (Escobar et al., 2020; Jørgensen & Ulloa Ulloa, 1994). This demarcation line, part of the Amotape–Huancabamba zone, has acted both as a dispersal barrier for montane species as well as a corridor for lowland species (Trénel et al., 2008; Weigend, 2002).

Understanding how tropical plants reacted to Pleistocene climatic fluctuations, that is whether they retracted into refugia (“dry‐refugia” hypothesis) or underwent down‐ and upslope migrations (“moist‐forest” hypothesis), remains a major conundrum (Carnaval et al., 2009, Valencia et al., 2010, Ramírez‐Barahona & Eguiarte, 2013, Escobar et al., 2020). If species retracted into small refugia, climatic instability through time should explain population genetic variation (Helmstetter et al., 2020). In our study, we found significant associations between genetic differentiation and climatic instability in five species (Table 1), but IBI was recovered as the factor best explaining variation in *F*_ST_ only in *M*. *tomentosa*. Indeed, modelling past climatic habitat suitability indicated that the cloud forest species *Ax*. *costaricensis*, *M*. *maxima*, *M*. *phlomoides* and *M*. *tomentosa* retained relatively continuous suitable habitats along the Central and Northern South American mountain ranges throughout the Pleistocene (Figure S3). These results support the “moist‐forest” hypothesis, with downslope and subsequent upslope migration during Pleistocene climatic fluctuations (Figure S14), and even range expansion in *M*. *phlomoides* (Figure S3c). Further, at the scale of our study localities, there was some evidence of habitat contraction during the LGM in *Ax*. *costaricensis* (continuously suitable around locality 4; Figure S6) and *M*. *maxima* (continuously suitable around localities 1, 2 and 5; Figure S7). This suggests the possibility for *in situ* persistence of these cloud‐forest species in part of the distribution range (without necessarily contraction into isolated refugia), a pattern documented for other montane Neotropical plant lineages (Ornelas et al., 2019, Hernández‐Langford et al., 2020). *M*. *sanguinea*, on the other hand, is the only species in our sample occurring in the high‐elevation cloud‐forest–Páramo ecotone. This species may have undergone more prominent refugial retraction in southern Ecuador and Peru (Figures S3 and S14). Indeed, our models indicate little connectivity among the southern Ecuadorian localities (south of the biogeographical barrier of the Amotape–Huancabamba zone) and markedly differentiated northern Ecuadorian locality during the LGM (Figure S7). Finally, in lowland bee‐pollinated *Ad*. *adscendens*, suitable habitats were probably extensive in lowland Amazonia during the LGM, with mostly continuously suitable habitats along the (eastern) coast of Central America (Figure S6; Pröhl et al., 2010). However, our sampling covered only parts of each species’ distribution range and to fully understand whether and where cloud forests or foothill forests may have acted as refugial areas, it will be necessary to sample localities across the full distribution range of each species (e.g., see approach in Escobar et al., 2020; Helmstetter et al., 2020). Such broad‐scale sampling will also be relevant to test more explicitly (i.e., using distance‐based redundancy analyses; He et al., 2013) whether genetic variation of species differing in pollination strategy responds differently to topographic and climatic factors.

Together, our results are highly valuable in adding to the limited data available on the diverse processes shaping population genetic differentiation of tropical plants, including differences in pollination strategy. While we highlight that a wider sampling across bee‐pollinated Merianieae is required to firmly establish the role of pollinators in promoting population differentiation, our result on stronger isolation among bee‐ than vertebrate‐pollinated populations suggests a critical role of pollinator mobility in shaping population‐level processes. Extrapolating to a macroevolutionary perspective, pollinator shifts are often invoked as “key innovations” spurring diversification (van der Niet et al., 2014). Potentially, pollinator shifts may also alter a population's susceptibility to isolation and, consequently, its potential for allopatric divergence (Kisel & Barraclough, 2010). Interestingly, in various Neotropical/Andean plant groups, shifts from bee to vertebrate (particularly hummingbird) pollination go hand in hand with increases in diversification rates (e.g., Lagomarsino et al., 2016, Serrano‐Serrano et al., 2017). This is somewhat counterintuitive, however, if vertebrates are expected to buffer isolating effects among populations (see Serrano‐Serrano et al., 2017 for a discussion on additional factors). Clearly, comparative studies, ideally focusing on small monophyletic plant complexes, and documenting both pollination ecology and population genetics of multiple populations across the landscape (e.g., Opedal et al., 2016), will be essential for resolving the relative contribution of pollinator shifts in spurring or limiting speciation through gene flow (Abrahamczyk et al., 2014; Kartzinel et al., 2013; Kisel et al., 2010). To date, we largely lack pollinator observations from multiple populations of the same plant species in the tropics, and hence know little about the variability of pollinator composition across a species’ distribution range (pollinator mosaic; Gowda & Kress, 2013). Within our own data set, we have such information for only a subset of all populations (Table S1). While we documented the same functional groups acting as pollinators in most populations, we documented effective hummingbird and rodent pollinators in southern Ecuadorian populations of *M*. *sanguinea*, and hummingbird and bat pollinators in northern Ecuador. Obtaining such natural history information, in addition to population genomic data, will be key to adding a more realistic understanding of the processes governing speciation. This will ultimately help in addressing broad‐scale questions such as how the tropics worldwide have become exceptionally biodiverse, but also in tracing similarities and differences in drivers of diversification across different tropical habitats and continents, namely Andean uplift in the Neotropics (Lagomarsino et al., 2016) and aridification in the African tropics (Couvreur et al., 2020).

## AUTHOR CONTRIBUTIONS

ASD, JS and OP conceived and designed the study, ASD and DFF performed fieldwork, ASD, JB and EMT conducted laboratory work, ASD and OP analysed the genomic data, ASD performed niche modelling and wrote the first draft; all authors contributed to revising the manuscript.

## Supporting information

Supplementary Material

## Data Availability

All new genomic sequence data have been uploaded to NCBI Short Reads Archive (BioProject ID PRJNA780082) and are accessible at: https://www.ncbi.nlm.nih.gov/bioproject/PRJNA780082. Input data for niche modelling and exemplary R‐scripts for *Ax*. *costaricensis* have been deposited on the public repository Phaidra and are available for download at: https://phaidra.univie.ac.at/o:1417080.
