# Supplementary material for: Population structure in Neotropical plants: Integrating pollination biology, topography and climatic niches
Source: Mol Ecol. 2022 Mar 2;31(8):2264–80. doi: 10.1111/mec.16403 (PMC9310734; doi:10.1111/mec.16403)
Supplement: Supplementary file 1 — Supplementary Material [file MEC-31-2264-s001.docx]

**Supplemental Information for:**

**Population structure in Neotropical plants: integrating pollination biology, topography and climatic niches**

Agnes S. Dellinger, Ovidiu Paun, Juliane Baar, Eva M. Temsch, Diana Fernández-Fernández, Jürg Schönenberger

**Table of Contents:**

| **Table S1.** **Details on sampling localities for each species** | Page 3 |
| --- | --- |
| **Table S2. Pairwise population genetic differentiation** | Page 5 |
| **Table S3.** Tukey-HSD post-hoc test | Page 6 |
| **Table S4. The average number of raw reads** | Page 6 |
| **Table S5. Number of pruned GBIF occurrences** | Page 7 |
| **Table S6: Evaluation of MaxEnt models** | Page 7 |
| **Table S7: Mantel’s tests on the four explanatory geographic distance matrices** | Page 8 |
| **Figure S1. GBIF occurrences of our six study species** | Page 9 |
| **Figure S2a: MESS *Ad. adscendens*** | Page 10 |
| **Figure S2b: MESS *M. maxima*** | Page 11 |
| **Figure S2c: MESS *M. phlomoides*** | Page 12 |
| **Figure S2d: MESS *M. sanguinea*** | Page 13 |
| **Figure S2e: MESS *M. tomentosa*** | Page 14 |
| **Figure S2f: MESS *A. costaricensis*** | Page 15 |
| **Figure S3a.** **Habitat resistance *Ad. adscendens*** | Page 16 |
| **Figure S3b.** **Habitat resistance *M. maxima*** | Page 17 |
| **Figure S3c.** **Habitat resistance *M. phlomoides*** | Page 18 |
| **Figure S3d.** **Habitat resistance *M. sanguinea*** | Page 19 |
| **Figure S3e.** **Habitat resistance *M. tomentosa*** | Page 20 |
| **Figure S3f.** **Habitat resistance *A. costaricensis*** | Page 21 |
| **Table S8. Nucleotide diversity** | Page 22 |
| **Table S9. Results from Kruskal-Wallis ANOVA** | Page 23 |
| **Table S10. Results from Dunn test** | Page 23 |
| **Table S11. Average (and standard deviation) heterozygosity** | Page 24 |
| **Table S12. Results from Dunn test** | Page 24 |
| **Table S13. Average (and standard deviation) inbreeding coefficients** | Page 26 |
| **Table S14. Mantel’s test results on genetic and geographic distances** | Page 26 |
| **Table S15. Significant differences in pairwise population genetic differentiation** | Page 27 |
| **Table S16. Significant differences in pairwise population genetic differentiation** | Page 29 |
| **Table S17. Pairwise comparison of differences in disparity** | Page 31 |
| **Table S18. Pairwise comparison of differences in disparity** | Page 33 |
| **Table S19. Results of Mantel’s tests IBD, IBR_Terrain_, IBR_Habitat_, IBI and IBE** | Page 34 |
| **Table S20. Results of Multiple Matrix Regression with Randomization** | Page 35 |
| **Table S21. Results of Generalized Dissimilarity Modelling** | Page 35 |
| **Figure S4. Per-individual inbreeding coefficient** | Page 37 |
| **Figure S5. Population genetic differentiation** | Page 38 |
| **Figure S6. Habitat resistance and least-cost paths in Costa Rica** | Page 40 |
| **Figure S7. Habitat resistance and least-cost paths in Ecuador** | Page 41 |
| **Figure S8.** **GDM-fitted I splines in *Ad. adscendens*** | Page 42 |
| **Figure S9.** **GDM-fitted I splines in *M. maxima*** | Page 43 |
| **Figure S10.** **GDM-fitted I splines in *M. phlomoides*** | Page 44 |
| **Figure S11.** **GDM-fitted I splines in *M. sanguinea*** | Page 45 |
| **Figure S12.** **GDM-fitted I splines in *M. tomentosa*** | Page 46 |
| **Figure S13.** **GDM-fitted I splines in *A. costaricensis*** | Page 47 |
| **Figure S14. Changes in elevational distribution of the six study species** | Page 48 |

**Methods**

**Table S1.** **Details on sampling localities for each species**, coordinates, maximum distance across which individuals were sampled, and number of individuals sampled from each locality. Field trips to Costa Rica were performed in 2015, field trips to Ecuador in 2016 and 2017. We also indicate for which localities we have observed pollinators, details may be found in Dellinger et al. 2019, 2021. Note that “?” indicates lack of nocturnal observations, “hb” indicates “hummingbirds”.

| **Species** | **Locality** | **Locality information** | **Pollinator observations** | **Longitude** | **Latitude** | **Elevation (masl)** | **Maximum distance (m)** | **No of individuals** |
| --- | --- | --- | --- | --- | --- | --- | --- | --- |
| *Adelobotrys adscendens* | 1 | Costa Rica, forest along rio Peñas Blancas, Monteverde | - | -84.7399 | 10.3036 | 967 | 1195 | 10 |
|  | 2 | Costa Rica, Volcán Tenorio National Park | - | -84.9937 | 10.7045 | 834 | 1149 | 17 |
|  | 3 | Costa Rica, Braulio Carillo National Park, Río Sucio | - | -83.9447 | 10.1538 | 456 | 293 | 13 |
|  | 4 | Costa Rica, Piedras Blancas National Park, around Tropical Field Station La Gamba | small bees | -83.205 | 8.6999 | 130 | 776 | 16 |
|  | 5 | Costa Rica, Refugio Forestal Golfito (Cerro Torre) | - | -83.1641 | 8.6507 | 387 | 701 | 12 |
|  | 6 | Costa Rica, Potrero Grande, forest patch along roadside | - | -83.0939 | 9.0703 | 845 | 187 | 13 |
| *Meriania maxima* | 1 | Ecuador, Pichincha Province, Bellavista Cloudforest Reserve | large bees | -78.6794 | -0.0091 | 2002 | 1261 | 10 |
|  | 2 | Ecuador, Pichincha Province, forest along roadside | - | -78.6332 | 0.0244 | 2184 | 290 | 7 |
|  | 3 | Ecuador, Carchi Province, Reserva Drácula | - | -78.0449 | 0.8309 | 2500 | 2101 | 12 |
|  | 4 | Ecuador, Carchi Province, Reserva Drácula | - | -78.2253 | 0.8808 | 1974 | 1682 | 11 |
|  | 5 | Ecuador, Pichincha Province, forest on road to Mindo | large bees | -78.7605 | -0.0317 | 1650 | 801 | 11 |
| *Meriania phlomoides* | 1 | Costa Rica, Los Quetzales National Park | - | -83.8154 | 9.543 | 2208 | 996 | 13 |
|  | 2 | Costa Rica, Monteverde/Cerro Amigos | hb/bat | -84.7995 | 10.3244 | 1769 | 2709 | 14 |
|  | 3 | Costa Rica, Reserva Cerro Dantas | hb/bat | -84.0593 | 10.0954 | 2065 | 632 | 19 |
|  | 4 | Costa Rica, Chirripó National Park | - | -83.5664 | 9.4632 | 2161 | 1063 | 15 |
|  | 5 | Costa Rica, La Amistad National Park | - | -83.0651 | 9.1373 | 2036 | 286 | 15 |
|  | 6 | Costa Rica, Braulio Carillo National Park | - | -84.0261 | 10.0662 | 1888 | 238 | 14 |
| *Meriania sanguinea** | 1 | Ecuador, Loja Province, Podocarpus National Park, Cajanuma | hb/rodent | -79.1688 | -4.1167 | 2822 | 851 | 13 |
|  | 2 | Ecuador, Loja Province, Podocarpus National Park, Cerro Toledo | hb/? | -79.12 | -4.384 | 2985 | 1805 | 10 |
|  | 3 | Ecuador, Loja Province, páramo around El Tiro pass | hb/? | -79.1431 | -3.9863 | 2712 | 616 | 11 |
|  | 4 | Ecuador, Carchi Province, Reserva Guanderas | hb/bat | -77.7032 | 0.5918 | 3405 | 528 | 12 |
|  | 5 | Ecuador, Loja Province, Yacuri | - | -79.4111 | -4.7722 | 3160 | 488 | 12 |
|  | 6 | Ecuador, Zamora-Chinchipe Province, Tapichalaca Reserve | - | -79.1321 | -4.4953 | 2485 | 414 | 12 |
| *Meriania tomentosa* | 1 | Ecuador, Pichincha Province, Bellavista Cloudforest Reserve | hb/bat | -78.6813 | -0.0152 | 2002 | 609 | 9 |
|  | 2 | Ecuador, Loja Province, Podocarpus National Park, Cajanuma | hb/bat | -79.1683 | -4.117 | 2822 | 597 | 10 |
|  | 3 | Ecuador, Loja Province, páramo around El Tiro pass | - | -79.1397 | -3.9796 | 2791 | 644 | 10 |
|  | 4 | Ecuador, Pichincha Province, forest along roadside | hb/? | -78.6329 | 0.0238 | 2184 | 216 | 12 |
|  | 5 | Ecuador, Zamora-Chinchipe Province, Tapichalaca Reserve | - | -79.1307 | -4.4942 | 2485 | 547 | 11 |
| *Axinaea costaricensis* | 1 | Costa Rica, Reserva Cerro Dantas | - | -84.0601 | 10.0939 | 2065 | 2001 | 19 |
|  | 2 | Costa Rica, forest around Truchas Selva Madre | passerine | -83.8766 | 9.6789 | 2506 | 935 | 16 |
|  | 3 | Costa Rica, Cerros de Escazú | passerine | -84.1456 | 9.8702 | 2157 | 3400 | 15 |
|  | 4 | Costa Rica, Chirripó National Park | - | -83.5404 | 9.4541 | 2608 | 116 | 13 |
|  | 5 | Costa Rica, Braulio Carillo National Park | - | -84.0249 | 10.0675 | 1810 | 222 | 15 |

* Note that we detected different functional groups acting as pollinators in localities of *M. sanguinea* (hummingbirds and rodents in Southern Ecuador, hummingbirds and bats in Northern Ecuador), and localities showing morphological differences across this 500 kilometre distance. Specifically, southern localities showed adaptations to rodent pollination (floral scent compounds, branches immersed in surrounding vegetation facilitating access to climbing rodents) and the northern localities to bat pollination (different floral scent compounds, flowers exposed from foliage, Dellinger et al. 2019b). We emphasize that this differentiation occurred across a much larger (500 km versus 20 km) distance than morphological differentiation in bee-pollinated *M. maxima*. Overall, we emphasize that such natural history data is very time-consuming and difficult to obtain since it requires extended fieldwork in remote areas and exact timing of fieldwork to match flowering (which can be challenging in tropical habitats), but absolutely crucial to better resolve the impact of pollinators on population differentiation.

**Table S2. Pairwise population genetic differentiation (F_ST_, lower sub-diagonal) and distance (km, upper sub-diagonal) between localities.**

| ***Ad. adscendens*** | **pop1** | **pop2** | **pop3** | **pop4** | **pop5** | **pop6** |
| --- | --- | --- | --- | --- | --- | --- |
| **pop1** |  | 52.3 | 88.7 | 244.7 | 251.7 | 226.4 |
| **pop2** | 0.366 |  | 130.0 | 296.1 | 303.2 | 257.8 |
| **pop3** | 0.369 | 0.189 |  | 180.1 | 187.1 | 151.9 |
| **pop4** | 0.566 | 0.497 | 0.492 |  | 7.1 | 42.8 |
| **pop5** | 0.609 | 0.523 | 0.522 | 0.058 |  | 47.0 |
| **pop6** | 0.661 | 0.578 | 0.578 | 0.148 | 0.177 |  |
|  |  |  |  |  |  |  |
| ***M. maxima*** | **pop1** | **pop2** | **pop3** | **pop4** | **pop5** |  |
| **pop1** |  | 6.3 | 116.7 | 110.6 | 9.4 |  |
| **pop2** | 0.018 |  | 110.6 | 105.0 | 15.5 |  |
| **pop3** | 0.228 | 0.251 |  | 20.8 | 124.3 |  |
| **pop4** | 0.230 | 0.244 | 0.283 |  | 117.18 |  |
| **pop5** | 0.036 | 0.036 | 0.296 | 0.283 |  |  |
|  |  |  |  |  |  |  |
|  |  |  |  |  |  |  |
| ***M. sanguinea*** | **pop1** | **pop2** | **pop3** | **pop4** | **pop5** | **pop6** |
| **pop1** |  | 30.1 | 14.7 | 545.6 | 77.3 | 42.1 |
| **pop2** | 0.061 |  | 44.1 | 572.3 | 53.7 | 12.4 |
| **pop3** | 0.031 | 0.052 |  | 531.000 | 91.900 | 56.300 |
| **pop4** | 0.315 | 0.293 | 0.273 |  | 622.800 | 584.500 |
| **pop5** | 0.093 | 0.065 | 0.082 | 0.316 |  | 43.500 |
| **pop6** | 0.098 | 0.063 | 0.088 | 0.316 | 0.069 |  |
|  |  |  |  |  |  |  |
| ***M. phlomoides*** | **pop1** | **pop2** | **pop3** | **pop4** | **pop5** | **pop6** |
| **pop1** |  | 138.3 | 66.7 | 28.7 | 93.8 | 62.3 |
| **pop2** | 0.129 |  | 85.0 | 165.4 | 231.2 | 89.4 |
| **pop3** | 0.097 | 0.113 |  | 88.4 | 152.1 | 4.9 |
| **pop4** | 0.073 | 0.138 | 0.109 |  | 65.8 | 83.6 |
| **pop5** | 0.193 | 0.223 | 0.190 | 0.144 |  | 147.300 |
| **pop6** | 0.127 | 0.144 | 0.037 | 0.120 | 0.202 |  |
|  |  |  |  |  |  |  |
| ***M. tomentosa*** | **pop1** | **pop2** | **pop3** | **pop4** | **pop5** |  |
| **pop1** |  | 456.8 | 441.3 | 6.9 | 497.8 |  |
| **pop2** | 0.179 |  | 15.5 | 461.7 | 41.9 |  |
| **pop3** | 0.191 | 0.044 |  | 20.8 | 124.3 |  |
| **pop4** | 0.046 | 0.195 | 0.200 |  | 117.200 |  |
| **pop5** | 0.216 | 0.080 | 0.088 | 0.240 |  |  |
|  |  |  |  |  |  |  |
| ***A. costaricensis*** | **pop1** | **pop2** | **pop3** | **pop4** | **pop5** |  |
| **pop1** |  | 50.1 | 26.5 | 90.9 | 4.8 |  |
| **pop2** | 0.088 |  | 36.3 | 44.5 | 46.0 |  |
| **pop3** | 0.079 | 0.083 |  | 80.8 | 25.5 |  |
| **pop4** | 0.143 | 0.146 | 0.165 |  | 86.2 |  |
| **pop5** | 0.105 | 0.119 | 0.092 | 0.207 |  |  |

**Table S3.** Tukey-HSD post-hoc test for pairwise comparisons in mapping rates between the different species when mapping all species to the common pseudo-reference. The mapping rate of *Ad. adscendens* (Aa) was significantly lower than in all other species and we hence decided to map this species to its own pseudo-reference. Aa – *Ad. adscendens*, Ac – *A. costaricensis*, Mm – *M. maxima*, Mp – *M. phlomoides*, Ms – *M. sanguinea*, Mt – *M. tomentosa*.

| **pairwise comparisons** | **diff** | **lwr** | **upr** | **p adj** |
| --- | --- | --- | --- | --- |
| **Ac-Aa** | 22.5133333 | 18.8855896 | 26.1410771 | *0.0000000* |
| **Mm-Aa** | 18.4983030 | 14.5027817 | 22.4938244 | *0.0000000* |
| **Mp-Aa** | 17.0971111 | 13.5947401 | 20.5994821 | *0.0000000* |
| **Ms-Aa** | 10.9656808 | 7.2479497 | 14.6834118 | *0.0000000* |
| **Mt-Aa** | 21.8570115 | 17.9235152 | 25.7905078 | *0.0000000* |
| **Mm-Ac** | -4.0150303 | -8.0415055 | 0.0114449 | 0.0511400 |
| **Mp-Ac** | -5.4162222 | -8.9538647 | -1.8785797 | *0.0002130* |
| **Ms-Ac** | -11.5476526 | -15.2986306 | -7.7966746 | *0.0000000* |
| **Mt-Ac** | -0.6563218 | -4.6212563 | 3.3086126 | 0.9970220 |
| **Mp-Mm** | -1.4011919 | -5.3150877 | 2.5127039 | 0.9094833 |
| **Ms-Mm** | -7.5326223 | -11.6403591 | -3.4248855 | *0.0000036* |
| **Mt-Mm** | 3.3587085 | -0.9452863 | 7.6627032 | 0.2243881 |
| **Ms-Mp** | -6.1314304 | -9.7612944 | -2.5015663 | *0.0000273* |
| **Mt-Mp** | 4.7599004 | 0.9093442 | 8.6104565 | *0.0059178* |
| **Mt-Ms** | 10.8913307 | 6.8438989 | 14.9387625 | *0.0000000* |

**Table S4. The average number of raw reads, mapping rate and coverage to the common reference** (used for *Axinaea costaricensis, Meriania maxima, M. phlomoides, M. sanguinea, M. tomentosa*) and the separate reference of more distantly-related *Adelobotrys adscendens*.

| **Species** | **average no raw reads** | **sd no raw reads** | **average mapping rate** | **sd mapping rate** | **average coverage** | **sd coverage** |
| --- | --- | --- | --- | --- | --- | --- |
| *Ad. adscendens*  common reference | 1589090.8 | 805425.2 | 0.318 | 0.058 | 9.026 | 4.644 |
| *Ad. adscendens*  own reference | 1589090.8 | 805425.2 | 0.351 | 0.070 | 16.359 | 10.001 |
| *A. costaricensis* | 2008191.2 | 737265.0 | 0.543 | 0.076 | 15.475 | 5.909 |
| *M. maxima* | 896203.4 | 393248.9 | 0.514 | 0.096 | 7.170 | 2.874 |
| *M. phlomoides* | 1839926.3 | 961480.5 | 0.489 | 0.080 | 13.482 | 7.711 |
| *M. sanguinea* | 907241.2 | 391229.1 | 0.429 | 0.090 | 6.450 | 2.967 |
| *M. tomentosa* | 736530.4 | 266044.3 | 0.538 | 0.059 | 5.861 | 2.254 |

**Table S5. Number of pruned GBIF occurrences kept for each species and elevation range** used for calculating TRI and present-day environmental niches (IBR_Habitat_). References to species-specific GBIF datasets are also given (downloaded on 20200909).

| **species** | **no of GBIF occurrences kept** | **elevation range for ENM** | **GBIF reference** |
| --- | --- | --- | --- |
| *Ad. adscendens* | 575 | 0 - 1700 | https://doi.org/10.15468/dl.m8kavy |
| *M. maxima* | 74 | 1132 - 2856 | https://doi.org/10.15468/dl.8qffg7 |
| *M. phlomoides* | 149 | 934 - 3012 | https://doi.org/10.15468/dl.9c7evg |
| *M. sanguinea* | 54 | 1433 - 3754 | https://doi.org/10.15468/dl.rmyqfp |
| *M. tomentosa* | 251 | 1204 - 3543 | https://doi.org/10.15468/dl.pyhepg |
| *A. costaricensis* | 86 | 1145 - 2986 | https://doi.org/10.15468/dl.543jp2 |

**Table S6: Evaluation of MaxEnt models (AUC, TSS for 80% training data, 20% testing data), and model validation through 4-fold spatial blocks** for the fine-scaled climatic data (30 arc sec, used to estimate IBR_Habitat_) and coarse-scaled climatic data (2.5 arc min, used to estimate IBI). AUC (Area under the receiver operating curve) larger than 0.75 indicates good model fit, AUC larger than 0.9 excellent fit; TSS larger than 0.4 indicates acceptable model, TSS larger than 0.8 indicates excellent model.

| **IBR_Habitat_** | **trainAUC** | **testAUC** | **trainTSS** | **testTSS** | **CV_AUC** | **CV_TSS** |
| --- | --- | --- | --- | --- | --- | --- |
| *A. adscendens* | 0.906 | 0.894 | 0.664 | 0.650 | 0.857 | 0.584 |
|  | 0.004 | 0.015 | 0.011 | 0.037 | 0.004 | 0.008 |
| *A. costaricensis* | 0.884 | 0.876 | 0.688 | 0.702 | 0.858 | 0.680 |
|  | 0.008 | 0.046 | 0.031 | 0.084 | 0.009 | 0.025 |
| *M. maxima* | 0.904 | 0.879 | 0.736 | 0.730 | 0.770 | 0.539 |
|  | 0.012 | 0.056 | 0.024 | 0.090 | 0.022 | 0.044 |
| *M. phlomoides* | 0.896 | 0.877 | 0.635 | 0.613 | 0.829 | 0.556 |
|  | 0.005 | 0.026 | 0.013 | 0.050 | 0.008 | 0.013 |
| *M. sanguinea* | 0.882 | 0.855 | 0.643 | 0.648 | 0.766 | 0.486 |
|  | 0.008 | 0.058 | 0.027 | 0.116 | 0.014 | 0.029 |
| *M. tomentosa* | 0.878 | 0.870 | 0.628 | 0.624 | 0.768 | 0.467 |
|  | 0.004 | 0.015 | 0.013 | 0.035 | 0.004 | 0.008 |
| **IBI** |  |  |  |  |  |  |
| *A. adscendens* | 0.904 | 0.891 | 0.665 | 0.648 | 0.827 | 0.563 |
|  | 0.003 | 0.013 | 0.009 | 0.033 | 0.021 | 0.032 |
| *A. costaricensis* | 0.977 | 0.977 | 0.856 | 0.882 | 0.950 | 0.849 |
|  | 0.001 | 0.009 | 0.009 | 0.034 | 0.024 | 0.058 |
| *M. maxima* | 0.934 | 0.929 | 0.782 | 0.809 | 0.816 | 0.659 |
|  | 0.007 | 0.032 | 0.023 | 0.071 | 0.040 | 0.069 |
| *M. phlomoides* | 0.955 | 0.949 | 0.788 | 0.788 | 0.865 | 0.629 |
|  | 0.002 | 0.013 | 0.011 | 0.032 | 0.021 | 0.049 |
| *M. sanguinea* | 0.957 | 0.948 | 0.832 | 0.836 | 0.853 | 0.681 |
|  | 0.003 | 0.019 | 0.014 | 0.039 | 0.036 | 0.052 |
| *M. tomentosa* | 0.975 | 0.974 | 0.902 | 0.901 | 0.949 | 0.867 |
|  | 0.001 | 0.005 | 0.005 | 0.018 | 0.004 | 0.013 |

**Table S7: Mantel’s tests on the four explanatory geographic distance matrices** (IBD – Euclidean distances between localities, IBR_Terrain_ – resistance surface based on TRI, IBR_Habitat_ - resistance surface based on habitat suitability estimated through environmental niche modelling, IBI – resistance surface on habitat suitability through current-mod-Holocene-LGM). In most species, the four matrices were significantly correlated (shown in red).

| **species** | **IBD - IBRTerrain** | | **IBD - IBRHabitat** | | **IBD - IBI** | | **IBRHabitat - IBRTerrain** | | **IBRHabitat - IBI** | | **IBRTerrain - IBI** | |
| --- | --- | --- | --- | --- | --- | --- | --- | --- | --- | --- | --- | --- |
|  | **R²** | ***p*** | **R²** | ***p*** | **R²** | ***p*** | **R²** | ***p*** | **R²** | ***p*** | **R²** | ***p*** |
| ***Ad. adscendens*** | 0.978 | *0.004* | 0.170 | 0.186 | 0.170 | 0.178 | 0.179 | 0.159 | 0.263 | 0.102 | *0.975* | *0.002* |
| ***M. maxima*** | 0.999 | *0.009* | *0.971* | *0.032* | *0.992* | *0.008* | *0.975* | *0.033* | *0.991* | *0.037* | *0.993* | *0.010* |
| ***M. phlomoides*** | 0.996 | *0.001* | *0.798* | *0.001* | *0.993* | *0.002* | *0.815* | *0.002* | *0.788* | *0.001* | *0.984* | *0.002* |
| ***M. sanguinea*** | 0.999 | *0.001* | *0.998* | *0.001* | *0.999* | *0.002* | *0.998* | *<0.001* | *0.999* | *<.0.001* | *0.999* | *0.001* |
| ***M. tomentosa*** | 0.999 | *0.009* | *0.999* | *0.008* | *0.999* | *0.008* | *0.999* | *0.009* | *0.999* | *0.009* | *0.999* | *0.008* |
| ***A. costaricensis*** | 0.960 | *0.009* | 0.296 | 0.148 | 0.296 | 0.142 | 0.503 | 0.136 | *0.759* | *0.031* | *0.858* | *0.010* |


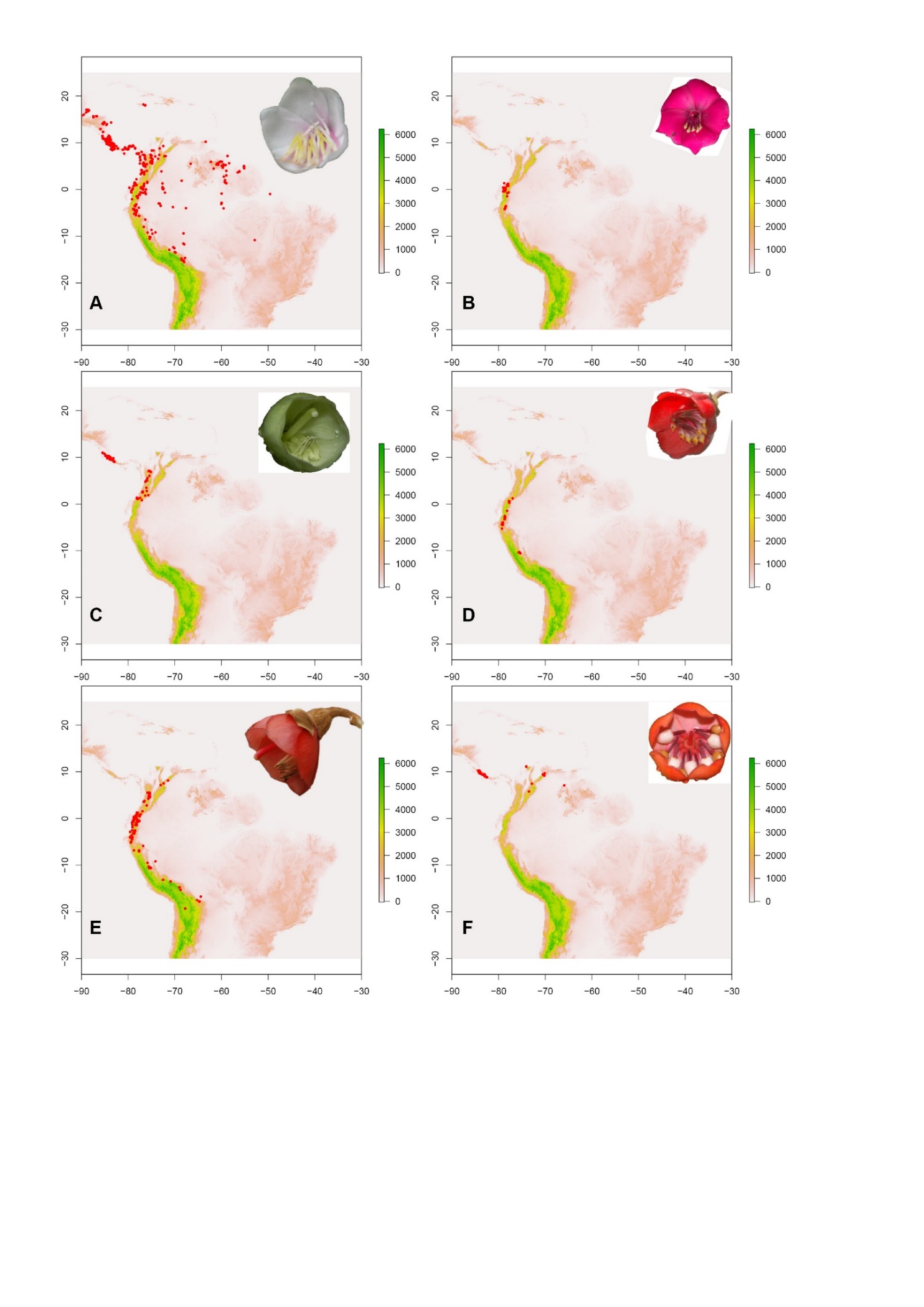


**Figure S1. GBIF occurrences of our six study species plotted on an elevation map of South America.** (A) *Adelobotrys adscendens* has a wide distribution through lowland rainforest areas and intermontane moist valleys both in Central America, the tropical Andes and the adjacent Amazon lowlands. (B) *Meriania maxima* has a relatively restricted distribution area in the cloud forests of the tropical Andes (Ecuador, Colombia). (C) *Meriania phlomoides* is a relatively common cloud forest species in the Central American mountain ranges and in Colombian cloud forests. (D) *Meriania sanguinea* is a relatively rare species occurring at the upper limits of cloud forests and the transition zone to high Andean Páramo grasslands in Southern Colombia, Ecuador and Norther Peru. (E) *Meriania tomentosa* is a relatively widely distributed, common species in cloud forests of Colombia, Ecuador and Peru. (F) *Axinaea costaricensis* occurs in upper montane cloud forests in Costa Rica and the Norther Colombian mountain ranges.

**Figure S2a: Histogram of Multivariate Environmental Similarity Surface (MESS)** for ***Adelobotrys adscendens***: models were trained on occurrence records plus 10000 random background values and projected into the past environments (three different circulation models); negative values indicate high dissimilarity, which limits the predictive power of the projection.

**Figure S2b: Histogram of Multivariate Environmental Similarity Surface (MESS)** for ***Meriania maxima***: models were trained on occurrence records plus 10000 random background values and projected into the past environments (three different circulation models); negative values indicate high dissimilarity, which limits the predictive power of the projection.

**Figure S2c: Histogram of Multivariate Environmental Similarity Surface (MESS)** for ***Meriania phlomoides***: models were trained on occurrence records plus 10000 random background values and projected into the past environments (three different circulation models); negative values indicate high dissimilarity, which limits the predictive power of the projection.

**Figure S2d: Histogram of Multivariate Environmental Similarity Surface (MESS)** for ***Meriania sanguinea***: models were trained on occurrence records plus 10000 random background values and projected into the past environments (three different circulation models); negative values indicate high dissimilarity, which limits the predictive power of the projection.

**Figure S2e: Histogram of Multivariate Environmental Similarity Surface (MESS)** for ***Meriania tomentosa***: models were trained on occurrence records plus 10000 random background values and projected into the past environments (three different circulation models); negative values indicate high dissimilarity, which limits the predictive power of the projection.

**Figure S2f: Histogram of Multivariate Environmental Similarity Surface (MESS)** for ***Axinaea costaricensis***: models were trained on occurrence records plus 10000 random background values and projected into the past environments (three different circulation models); negative values indicate high dissimilarity, which limits the predictive power of the projection.


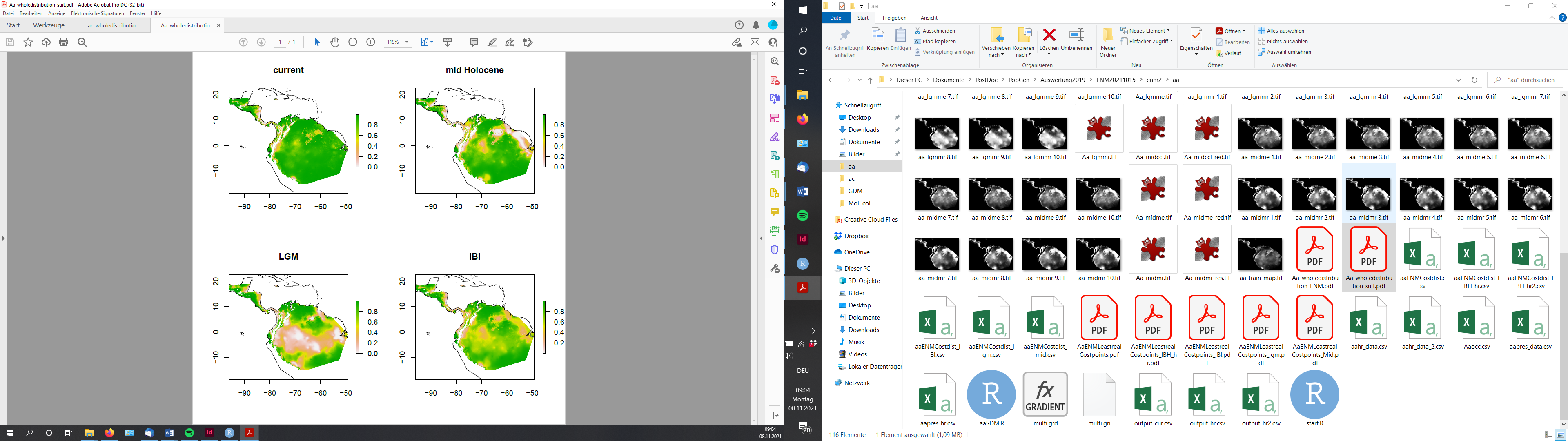


**Figure S3a.** **Habitat resistance for current climate, mid-Holocene (6 k years), the LGM (21 k years) and climatic instability (IBI) for the whole distribution range of lowland bee-pollinated *Ad. adscendens***, 0 indicates low habitat resistance (i.e. highly suitable habitat), 1 indicates high habitat resistance (i.e. unsuitable habitat). Habitat suitability changed markedly, with vast areas of lowland Amazonia suitable during LGM. We restricted niche models to buffered convex hulls spanning the known distribution range and masked out elevations above the current upper elevational range limits; these areas are indicated in white in the maps.


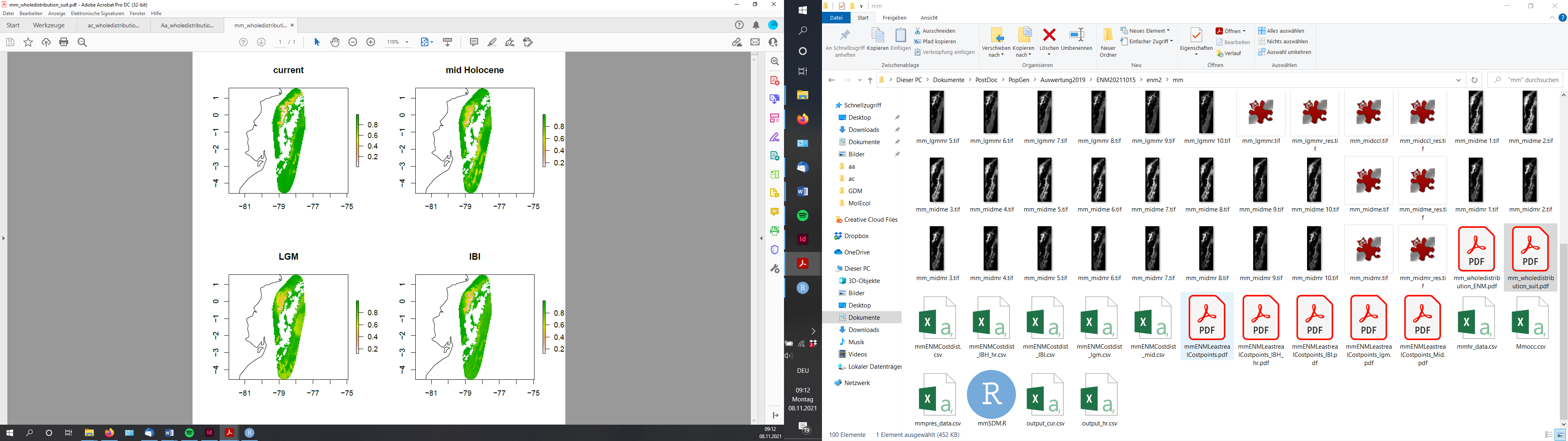


**Figure S3b.** **Habitat resistance for current climate, mid-Holocene (6 k years), the LGM (21 k years) and climatic instability (IBI) for the whole distribution range of montane bee-pollinated *M. maxima***, 0 indicates low habitat resistance (i.e. highly suitable habitat), 1 indicates high habitat resistance (i.e. unsuitable habitat). There were consistently suitable habitat conditions for *M. maxima* in the Western Andean cordillera in northern Ecuador and Southern Colombia. We restricted niche models to buffered convex hulls spanning the known distribution range and masked out elevations above the current upper elevational range limits; these areas are indicated in white in the maps.


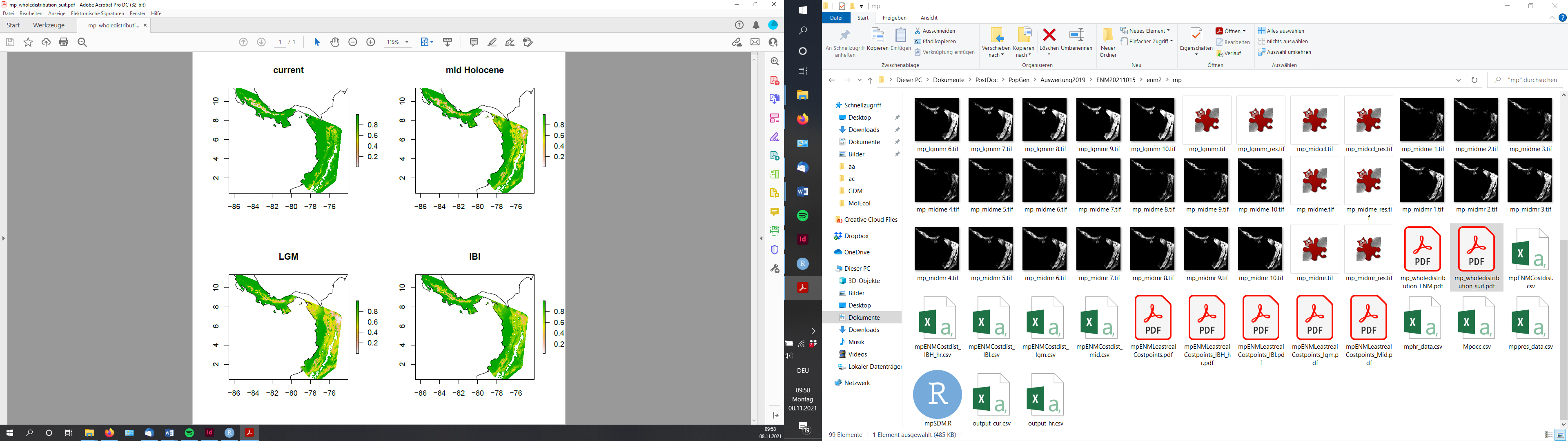


**Figure S3c.** **Habitat resistance for current climate, mid-Holocene (6 k years), the LGM (21 k years) and climatic instability (IBI) for the whole distribution range of montane bat-hummingbird-pollinated *M. phlomoides***, 0 indicates low habitat resistance (i.e. highly suitable habitat), 1 indicates high habitat resistance (i.e. unsuitable habitat). Our models indicate continued habitat suitability through time particularly in the montanous regions of Central America and the northern Andes of Colombia. We restricted niche models to buffered convex hulls spanning the known distribution range and masked out elevations above the current upper elevational range limits; these areas are indicated in white in the maps.


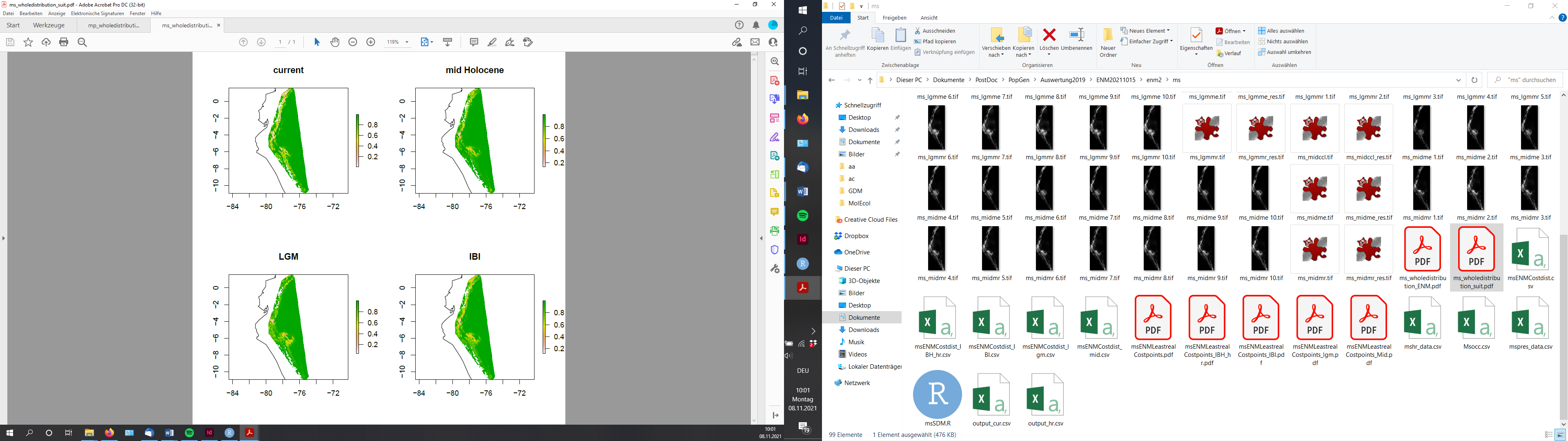


**Figure S3d.** **Habitat resistance for current climate, mid-Holocene (6 k years), the LGM (21 k years) and climatic instability (IBI) for the whole distribution range of montane hummingbird-rodent-bat-pollinated *M. sanguinea***, 0 indicates low habitat resistance (i.e. highly suitable habitat), 1 indicates high habitat resistance (i.e. unsuitable habitat). There was an overall reduction in suitable habitats during LGM for M. sanguinea, with a strong separation at the Amotape-Huancabamba zone. We restricted niche models to buffered convex hulls spanning the known distribution range and masked out elevations above the current upper elevational range limits; these areas are indicated in white in the maps.


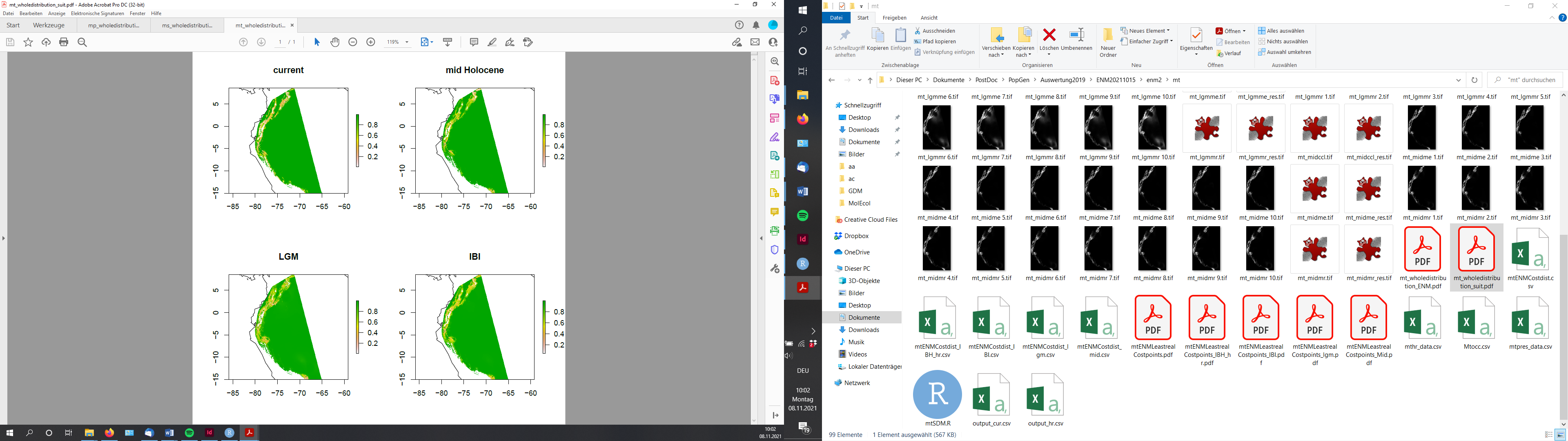


**Figure S3e.** **Habitat resistance for current climate, mid-Holocene (6 k years), the LGM (21 k years) and climatic instability (IBI) for the whole distribution range of montane hummingbird-bat-pollinated *M. tomentosa***, 0 indicates low habitat resistance (i.e. highly suitable habitat), 1 indicates high habitat resistance (i.e. unsuitable habitat). Our models indicate a relatively continuous distribution of suitable habitats in the Andean forests throughout the LGM. We restricted niche models to buffered convex hulls spanning the known distribution range and masked out elevations above the current upper elevational range limits; these areas are indicated in white in the maps.


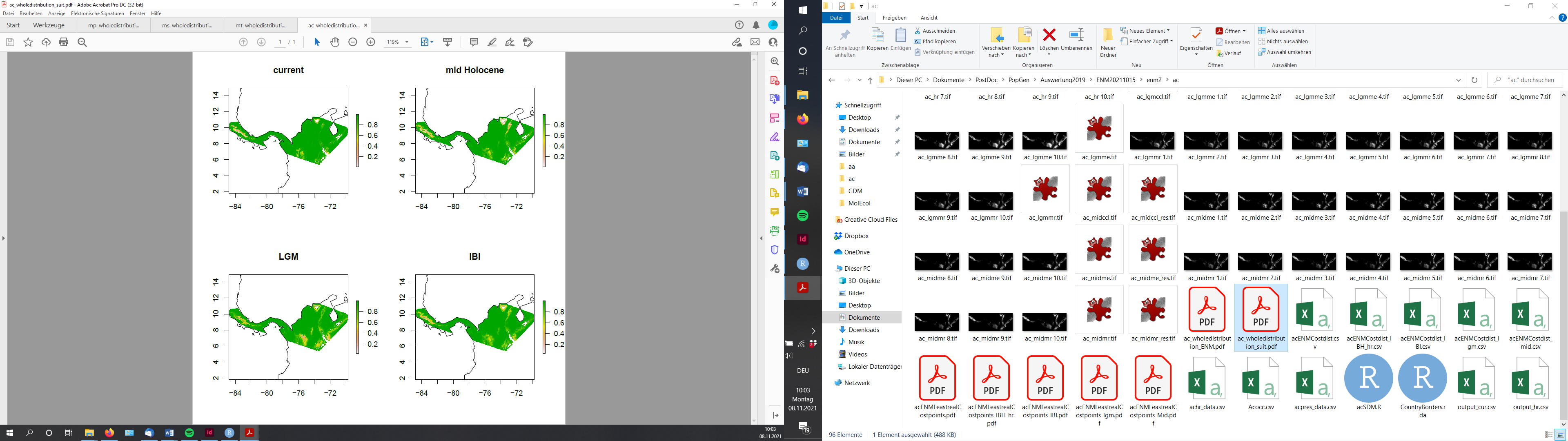


**Figure S3f.** **Habitat resistance for current climate, mid-Holocene (6 k years), the LGM (21 k years) and climatic instability (IBI) for the whole distribution range of montane passerine-pollinated *A. costaricensis***, 0 indicates low habitat resistance (i.e. highly suitable habitat), 1 indicates high habitat resistance (i.e. unsuitable habitat). Our models indicate an important role of the Central American montane forests and Northern Andean forests in Colombia for the distribution of *A. costaricensis* during the Holocene. We restricted niche models to buffered convex hulls spanning the known distribution range and masked out elevations above the current upper elevational range limits; these areas are indicated in white in the maps.

**Results**

**Table S8. Nucleotide diversity** (estimated by θ_π_) of the six study species, locality averages and standard deviation is given.

| **Species** | **Pollinator** | **Locality** | **Theta Pi (**θ_π)_ |
| --- | --- | --- | --- |
| *Ad. adscendens* | bee | 1 | 0.0006 (0.0026) |
|  |  | 2 | 0.0015 (0.0039) |
|  |  | 3 | 0.0014 (0.0037) |
|  |  | 4 | 0.003 (0.0051) |
|  |  | 5 | 0.003 (0.0053) |
|  |  | 6 | 0.0027 (0.005) |
| *M. maxima* | bee | 1 | 0.0057 (0.0063) |
|  |  | 2 | 0.0054 (0.0061) |
|  |  | 3 | 0.0051 (0.006) |
|  |  | 4 | 0.0044 (0.0055) |
|  |  | 5 | 0.0046 (0.006) |
| *M. phlomoides* | mv | 1 | 0.0033 (0.0066) |
|  |  | 2 | 0.0032 (0.0064) |
|  |  | 3 | 0.0036 (0.0073) |
|  |  | 4 | 0.0035 (0.007) |
|  |  | 5 | 0.0032 (0.0072) |
|  |  | 6 | 0.0032 (0.0064) |
| *M. sanguinea* | mv | 1 | 0.0056 (0.0061) |
|  |  | 2 | 0.006 (0.0061) |
|  |  | 3 | 0.0065 (0.0063) |
|  |  | 4 | 0.0044 (0.0059) |
|  |  | 5 | 0.0055 (0.0058) |
|  |  | 6 | 0.0053 (0.0056) |
| *M. tomentosa* | mv | 1 | 0.0073 (0.0075) |
|  |  | 2 | 0.0069 (0.0075) |
|  |  | 3 | 0.0067 (0.0077) |
|  |  | 4 | 0.0073 (0.0078) |
|  |  | 6 | 0.0056 (0.0067) |
| *A. costaricensis* | pass | 1 | 0.0024 (0.0058) |
|  |  | 2 | 0.0024 (0.0058) |
|  |  | 3 | 0.0024 (0.0059) |
|  |  | 4 | 0.0018 (0.0055) |
|  |  | 5 | 0.0022 (0.0058) |

**Table S9. Results from Kruskal-Wallis ANOVA testing for significant difference in nucleotide diversity and heterozygosity** among localities of the six study species.

|  | **nucleotide diverstity** | | | **heterozygosity** | | |
| --- | --- | --- | --- | --- | --- | --- |
| **species** | **X²** | **DF** | **p** | **X²** | **DF** | **p** |
| ***Ad. adscendens*** | 34497.6 | 5 | <0.001 | 20.5 | 5 | <0.001 |
| ***M. maxima*** | 4665.0 | 4 | <0.001 | 11.2 | 4 | 0.02 |
| ***M. phlomoides*** | 5056.7 | 5 | <0.001 | 12.4 | 5 | 0.03 |
| ***M. sanguinea*** | 9723.4 | 5 | <0.001 | 11.1 | 5 | 0.05 |
| ***M. tomentosa*** | 3902.3 | 4 | <0.001 | 18.0 | 4 | <0.001 |
| ***A. costaricensis*** | 6482.9 | 4 | <0.001 | 1.6 | 4 | 0.81 |

**Table S10. Results from Dunn test on significant differences in nucleotide diversity among localities**, lower sub-diagonal: Z-value, upper sub-diagonal: p-value; ns – not significant, * p < 0.05, ** p < 0.01.

| ***Ad. adscendens*** | | |  |  |  |  |
| --- | --- | --- | --- | --- | --- | --- |
|  | **pop1** | **pop2** | **pop3** | **pop4** | **pop5** | **pop6** |
| **pop1** |  | ** | ** | ** | ** | ** |
| **pop2** | -90.11 |  | ** | ** | ** | ** |
| **pop3** | -64.31 | 25.79 |  | ** | ** | ** |
| **pop4** | -160.87 | -70.76 | -96.55 |  | ** | ** |
| **pop5** | -140.50 | -50.39 | -76.19 | 20.37 |  | ** |
| **pop6** | -124.44 | -34.33 | -60.13 | 36.43 | 16.06 |  |
|  |  |  |  |  |  |  |
| ***M. maxima*** | |  |  |  |  |  |
|  | **pop1** | **pop2** | **pop3** | **pop4** | **pop5** |  |
| **pop1** |  | ** | ** | ** | ** |  |
| **pop2** | 6.85 |  | ** | ** | ** |  |
| **pop3** | 23.51 | 16.65 |  | ** | ** |  |
| **pop4** | 51.92 | 45.07 | 28.41 |  | ** |  |
| **pop5** | 50.77 | 43.92 | 27.27 | -1.15 |  |  |
|  |  |  |  |  |  |  |
| ***M. phlomoides*** | |  |  |  |  |  |
|  | **pop1** | **pop2** | **pop3** | **pop4** | **pop5** | **pop6** |
| **pop1** |  | ns | ** | ** | ** | ** |
| **pop2** | -1.79 |  | ** | ** | ** | ** |
| **pop3** | -11.39 | -9.60 |  | ** | ** | * |
| **pop4** | -18.94 | -17.15 | -7.55 |  | ** | ** |
| **pop5** | 42.85 | 44.64 | 54.23 | 61.78 |  | ** |
| **pop6** | -14.25 | -12.46 | -2.86 | 4.68 | -57.10 |  |
|  |  |  |  |  |  |  |
| ***M. sanguinea*** | |  |  |  |  |  |
|  | **pop1** | **pop2** | **pop3** | **pop4** | **pop5** | **pop6** |
| **pop1** |  | ** | ** | ** | ns | ** |
| **pop2** | -15.32 |  | ** | ** | ** | ** |
| **pop3** | -35.70 | -20.38 |  | ** | ** | ** |
| **pop4** | 57.64 | 72.96 | 93.34 |  |  | ** |
| **pop5** | 2.18 | 17.50 | 37.88 | -55.46 |  | ** |
| **pop6** | 8.79 | 24.11 | 44.49 | -48.85 | 6.61 |  |
|  |  |  |  |  |  |  |
| ***M. tomentosa*** | |  |  |  |  |  |
|  | **pop1** | **pop2** | **pop3** | **pop4** | **pop5** |  |
| **pop1** |  | ** | ** | ** | ** |  |
| **pop2** | 13.92 |  | ** | ** | ** |  |
| **pop3** | 25.24 | 11.32 |  | ** | ** |  |
| **pop4** | 6.27 | -7.65 | -18.97 |  | ** |  |
| **pop5** | 56.04 | 42.11 | 30.79 | 49.77 |  |  |
|  |  |  |  |  |  |  |
| ***A. costaricensis*** | |  |  |  |  |  |
|  | **pop1** | **pop2** | **pop3** | **pop4** | **pop5** |  |
| **pop1** |  | ** | ** | ** | ** |  |
| **pop2** | -12.56 |  | ns | ** | ** |  |
| **pop3** | -11.59 | 0.96 |  | ** | ** |  |
| **pop4** | 53.14 | 65.70 | 64.74 |  | ** |  |
| **pop5** | 27.72 | 40.27 | 39.31 | -25.43 |  |  |

**Table S11. Average (and standard deviation) heterozygosity** per locality of the six study species.

| **species** | **pop 1** | **pop 2** | **pop 3** | **pop 4** | **pop 5** | **pop 6** |
| --- | --- | --- | --- | --- | --- | --- |
| ***Ad. adscendens*** | 0.009 (0.004) | 0.016 (0.008) | 0.016 (0.007) | 0.018 (0.008) | 0.024 (0.01) | 0.013 (0.007) |
| ***M. maxima*** | 0.014 (0.001) | 0.015 (0.007) | 0.013 (0.003) | 0.012 (0.003) | 0.012 (0.002) |  |
| ***M. phlomoides*** | 0.013 (0.004) | 0.013 (0.004) | 0.017 (0.003) | 0.014 (0.004) | 0.015 (0.003) | 0.014 (0.003) |
| ***M. sanguinea*** | 0.014 (0.002) | 0.015 (0.003) | 0.016 (0.003) | 0.013 (0.002) | 0.013 (0.002) | 0.013 (0.004) |
| ***M. tomentosa*** | 0.019 (0.002) | 0.018 (0.001) | 0.019 (0.002) | 0.017 (0.001) | 0.015 (0.002) |  |
| ***A. costaricensis*** | 0.011 (0.004) | 0.011 (0.003) | 0.011 (0.004) | 0.010 (0.002) | 0.011 (0.002) |  |

**Table S12. Results from Dunn test on significant differences in heterozygosity among localities**, lower sub-diagonal: Z-value, upper sub-diagonal: p-value; ns – not significant, * p < 0.05, ** p < 0.01.

| ***Ad. adscendens*** | | |  |  |  |  |
| --- | --- | --- | --- | --- | --- | --- |
|  | **pop1** | **pop2** | **pop3** | **pop4** | **pop5** | **pop6** |
| **pop1** |  | ns | ns | * | * | ns |
| **pop2** | -2,44 |  | ns | ns | ns | ns |
| **pop3** | -2,37 | -0,06 |  | ns | ns | ns |
| **pop4** | -3,19 | -0,90 | -0,78 |  | ns | ns |
| **pop5** | -4,15 | -2,13 | -195,00 | -1,28 |  | * |
| **pop6** | -1,50 | 0,92 | 0,93 | 1,75 | 2,86 |  |
|  |  |  |  |  |  |  |
| ***M. maxima*** | |  |  |  |  |  |
|  | **pop1** | **pop2** | **pop3** | **pop4** | **pop5** |  |
| **pop1** |  | ns | ns | ** | ns |  |
| **pop2** | 1,13 |  | ns | ns | ns |  |
| **pop3** | 2,09 | 0,71 |  | ns | ns |  |
| **pop4** | 3,12 | 1,63 | 1,08 |  | ns |  |
| **pop5** | 2,47 | 1,08 | 0,44 | -0,61 |  |  |
|  |  |  |  |  |  |  |
| ***M. phlomoides*** | |  |  |  |  |  |
|  | **pop1** | **pop2** | **pop3** | **pop4** | **pop5** | **pop6** |
| **pop1** |  | ns | * | ns | ns | ns |
| **pop2** | -0,11 |  | ns | ns | ns | ns |
| **pop3** | -2,91 | -2,85 |  | ns | ns | ns |
| **pop4** | -0,70 | -0,60 | 2,26 |  | ns | ns |
| **pop5** | -1,46 | -1,38 | 1,43 | -0,79 |  | ns |
| **pop6** | -1,06 | -0,97 | 1,81 | -0,38 | 0,39 |  |
|  |  |  |  |  |  |  |
| ***M. sanguinea*** | |  |  |  |  |  |
|  | **pop1** | **pop2** | **pop3** | **pop4** | **pop5** | **pop6** |
| **pop1** |  | ns | ns | ns | ns | ns |
| **pop2** | -0,32 |  | ns | ns | ns | ns |
| **pop3** | -0,78 | -0,44 |  | ns | ns | ns |
| **pop4** | 1,58 | 1,82 | 2,27 |  | ns | ns |
| **pop5** | 1,58 | 1,83 | 2,28 | 0,01 |  | ns |
| **pop6** | 1,56 | 1,81 | 2,26 | -0,01 | -0,02 |  |
|  |  |  |  |  |  |  |
| ***M. tomentosa*** | |  |  |  |  |  |
|  | **pop1** | **pop2** | **pop3** | **pop4** | **pop5** |  |
| **pop1** |  | ns | ns | ns | * |  |
| **pop2** | -0,27 |  | ns | ns | ** |  |
| **pop3** | -0,64 | -0,38 |  | ns | ** |  |
| **pop4** | 1,12 | 1,44 | 1,84 |  | ns |  |
| **pop5** | 2,93 | 3,3 | 3,69 | 1,97 |  |  |
|  |  |  |  |  |  |  |
| ***A. costaricensis*** | |  |  |  |  |  |
|  | **pop1** | **pop2** | **pop3** | **pop4** | **pop5** |  |
| **pop1** |  | ns | ns | ns | ns |  |
| **pop2** | -0,27 |  | ns | ns | ns |  |
| **pop3** | -0,27 | -0,01 |  | ns | ns |  |
| **pop4** | 0,60 | 0,82 | 0,81 |  | ns |  |
| **pop5** | -0,7 | -0,42 | -0,41 | -1,21 |  |  |

**Table S13. Average (and standard deviation) inbreeding coefficients (F) per locality of the six study species.**

| **species** | **pop 1** | **pop 2** | **pop 3** | **pop 4** | **pop 5** | **pop 6** |
| --- | --- | --- | --- | --- | --- | --- |
| *Ad. adscendens* | 0.113 (0.146) | 0.034 (0.122) | 0.012 (0.044) | 0.001 (0.004) | 0.000 | 0.048 (0.125) |
| *M. maxima* | 0.000 | 0.000 | 4.5e-6 (1.6e-5) | 0.000 | 0.000 |  |
| *M. phlomoides* | 0.000 | 9.3e-7 (3.5e-6) | 0.000 | 4.3e-6 (1.7e-5) | 0.000 | 0.000 |
| *M. sanguinea* | 0.000 | 0.000 | 0.000 | 0.006 | 0.000 | 0.000 |
| *M. tomentosa* | 0.000 | 0.000 | 0.000 | 0.000 | 0.000 |  |
| *A. costaricensis* | 0.000 | 0.000 | 10e-6 (3.8e-6) | 0.000 | 0.000 |  |

**Table S14. Mantel’s test results on genetic and geographic distances among individuals within localities,** significant p-values are given in bolt.

| **species** | **locality** | **R²** | **p-value** |
| --- | --- | --- | --- |
| ***Ad. adscendens*** | **1** | **0.420** | ***0.009*** |
|  | 2 | 0.009 | *0.399* |
|  | **3** | **0.374** | ***0.022*** |
|  | 4 | -0.008 | *0.485* |
|  | 5 | 0.130 | *0.147* |
|  | **6** | **0.288** | ***0.01*** |
| ***M. maxima*** | **1** | **0.593** | ***0.007*** |
|  | 2 | 0.068 | *0.326* |
|  | 3 | -0.088 | *0.688* |
|  | **4** | **0.182** | ***0.025*** |
|  | 5 | -0.013 | *0.492* |
| *M. phlomoides* | 1 | -0.154 | *0.845* |
|  | 2 | -0.228 | *0.906* |
|  | 3 | -0.068 | *0.672* |
|  | 4 | -0.020 | *0.601* |
|  | **5** | **0.235** | ***0.007*** |
|  | 6 | 0.273 | *0.066* |
| *M. tomentosa* | 1 | -0.091 | *0.582* |
|  | 2 | -0.224 | *0.853* |
|  | 3 | 0.126 | *0.256* |
|  | **4** | **0.174** | ***0.02*** |
|  | 5 | 0.246 | *0.154* |
| *M. sanguinea* | 1 | 0.011 | *0.464* |
|  | 2 | -0.326 | *0.99* |
|  | 3 | 0.055 | *0.358* |
|  | 4 | 0.133 | *0.288* |
|  | 5 | 0.163 | *0.089* |
|  | 6 | 0.277 | *0.102* |
| *A. costaricensis* | 1 | -0.149 | *0.834* |
|  | **2** | **0.434** | ***0.011*** |
|  | 3 | 0.061 | *0.288* |
|  | 4 | 0.049 | *0.354* |
|  | 5 | 0.008 | *0.441* |

**Table S15. Significant differences in pairwise population genetic differentiation** (estimated from covariance matrix) of the different species. *Ad. adscendens*: F 6.88, R² 0.32, df = 5, p < 0.01, *M. maxima*: F 9.22, R² 0.44, df = 4, p < 0.01, *M. phlomoides*: F 5.4, R² 0.24, df = 5, p < 0.01, *M. sanguinea*: F 6.13, R² 0.32, df = 5, p < 0.01, *M. tomentosa*: F 5.50, R² 0.32, df = 4, p < 0.01, *A. costaricensis* F 2.99, R² 0.14, df = 4, p < 0.01. Locality pairs are indicated, as well as Sums of squares, F-values, R², and adjusted p-values (multiple comparisons); significant pairwise comparisons are highlighted in bold.

| **species** | **pairs** | **SumsOfSqs** | **F** | **R²** | **adj. p-value** |
| --- | --- | --- | --- | --- | --- |
| ***Ad. adscendens*** | **Aa1 vs Aa2** | **0.036** | **8.835** | **0.261** | **0.030** |
|  | **Aa1 vs Aa3** | **0.035** | **10.712** | **0.338** | **0.030** |
|  | **Aa1 vs Aa4** | **0.098** | **23.772** | **0.498** | **0.015** |
|  | **Aa1 vs Aa5** | **0.139** | **35.908** | **0.642** | **0.015** |
|  | **Aa1 vs Aa6** | **0.078** | **25.128** | **0.545** | **0.015** |
|  | Aa2 vs Aa3 | 0.004 | 0.785 | 0.027 | 1.000 |
|  | **Aa2 vs Aa4** | **0.065** | **12.735** | **0.291** | **0.015** |
|  | **Aa2 vs Aa5** | **0.074** | **14.595** | **0.351** | **0.015** |
|  | **Aa2 vs Aa6** | **0.071** | **15.756** | **0.360** | **0.015** |
|  | **Aa3 vs Aa4** | **0.049** | **10.314** | **0.276** | **0.015** |
|  | **Aa3 vs Aa5** | **0.061** | **13.137** | **0.364** | **0.015** |
|  | **Aa3 vs Aa6** | **0.055** | **14.012** | **0.369** | **0.015** |
|  | Aa4 vs Aa5 | 0.023 | 4.466 | 0.147 | 0.480 |
|  | Aa4 vs Aa6 | 0.009 | 1.989 | 0.069 | 1.000 |
|  | **Aa5 vs Aa6** | **0.050** | **11.328** | **0.330** | **0.030** |
| ***M. maxima*** | Mm_1 vs Mm_2 | 0.002 | 0.575 | 0.037 | 1.00 |
|  | **Mm_1 vs Mm_3** | **0.119** | **80.985** | **0.802** | **0.01** |
|  | **Mm_1 vs Mm_4** | **0.081** | **74.814** | **0.789** | **0.01** |
|  | Mm_1 vs Mm_5 | 0.005 | 3.047 | 0.138 | 0.40 |
|  | **Mm_2 vs Mm_3** | **0.103** | **36.106** | **0.680** | **0.01** |
|  | **Mm_2 vs Mm_4** | **0.061** | **25.460** | **0.600** | **0.01** |
|  | Mm_2 vs Mm_5 | 0.002 | 0.628 | 0.038 | 1.00 |
|  | **Mm_3 vs Mm_4** | **0.106** | **85.340** | **0.795** | **0.01** |
|  | **Mm_3 vs Mm_5** | **0.168** | **100.616** | **0.827** | **0.01** |
|  | **Mm_4 vs Mm_5** | **0.107** | **82.229** | **0.797** | **0.01** |
| ***M. phlomoides*** | Mp1-Mp2 | 0.009 | 1.519 | 0.057 | 1.000 |
|  | Mp1-Mp3 | 0.035 | 5.554 | 0.156 | 0.195 |
|  | Mp1-Mp4 | 0.004 | 0.646 | 0.024 | 1.000 |
|  | **Mp1-Mp5** | **0.051** | **10.283** | **0.283** | **0.015** |
|  | Mp1-Mp6 | 0.007 | 1.335 | 0.051 | 1.000 |
|  | Mp2-Mp3 | 0.035 | 6.007 | 0.162 | 0.105 |
|  | Mp2-Mp4 | 0.013 | 2.600 | 0.088 | 1.000 |
|  | **Mp2-Mp5** | **0.056** | **12.651** | **0.319** | **0.015** |
|  | Mp2-Mp6 | 0.012 | 2.345 | 0.083 | 1.000 |
|  | Mp3-Mp4 | 0.034 | 5.969 | 0.157 | 0.105 |
|  | Mp3-Mp5 | 0.033 | 6.505 | 0.169 | 0.060 |
|  | Mp3-Mp6 | 0.020 | 3.555 | 0.103 | 0.660 |
|  | **Mp4-Mp5** | **0.036** | **8.602** | **0.235** | **0.030** |
|  | Mp4-Mp6 | 0.008 | 1.673 | 0.058 | 1.000 |
|  | **Mp5-Mp6** | **0.041** | **10.116** | **0.273** | **0.015** |
| ***M. sanguinea*** | Ms1 vs Ms2 | 0.003 | 0.743 | 0.033 | 1.000 |
|  | Ms1 vs Ms3 | 0.002 | 0.350 | 0.016 | 1.000 |
|  | **Ms1 vs Ms4** | **0.140** | **40.468** | **0.638** | **0.015** |
|  | Ms1 vs Ms5 | 0.009 | 2.136 | 0.085 | 1.000 |
|  | Ms1 vs Ms6 | 0.010 | 2.084 | 0.083 | 1.000 |
|  | Ms2 vs Ms3 | 0.003 | 0.704 | 0.034 | 1.000 |
|  | **Ms2 vs Ms4** | **0.126** | **41.712** | **0.665** | **0.015** |
|  | Ms2 vs Ms5 | 0.004 | 0.953 | 0.043 | 1.000 |
|  | Ms2 vs Ms6 | 0.004 | 0.851 | 0.039 | 1.000 |
|  | **Ms3 vs Ms4** | **0.104** | **21.960** | **0.511** | **0.015** |
|  | Ms3 vs Ms5 | 0.011 | 1.954 | 0.085 | 1.000 |
|  | Ms3 vs Ms6 | 0.012 | 1.860 | 0.081 | 1.000 |
|  | **Ms4 vs Ms5** | **0.153** | **40.051** | **0.645** | **0.015** |
|  | **Ms4 vs Ms6** | **0.150** | **33.629** | **0.605** | **0.015** |
|  | Ms5 vs Ms6 | 0.003 | 0.528 | 0.023 | 1.000 |
| ***M. tomentosa*** | **Mt1 vs Mt2** | **0.072** | **112.156** | **0.868** | **0.01** |
|  | **Mt1 vs Mt3** | **0.071** | **103.136** | **0.858** | **0.01** |
|  | Mt1 vs Mt4 | 0.002 | 3.232 | 0.145 | 0.12 |
|  | **Mt1 vs Mt6** | **0.086** | **125.046** | **0.874** | **0.01** |
|  | Mt2 vs Mt3 | 0.001 | 1.375 | 0.071 | 1.00 |
|  | **Mt2 vs Mt4** | **0.098** | **187.375** | **0.904** | **0.01** |
|  | **Mt2 vs Mt6** | **0.003** | **4.400** | **0.188** | **0.02** |
|  | **Mt3 vs Mt4** | **0.098** | **171.929** | **0.896** | **0.01** |
|  | **Mt3 vs Mt6** | **0.004** | **5.349** | **0.220** | **0.02** |
|  | **Mt4 vs Mt6** | **0.117** | **205.551** | **0.907** | **0.01** |
| ***A. costaricensis*** | Ac_1 vs Ac_2 | 0.005 | 0.274 | 0.008 | 1 |
|  | Ac_1 vs Ac_3 | 0.005 | 0.296 | 0.009 | 1 |
|  | Ac_1 vs Ac_4 | 0.012 | 0.746 | 0.024 | 1 |
|  | Ac_1 vs Ac_5 | 0.014 | 0.983 | 0.030 | 1 |
|  | Ac_2 vs Ac_3 | 0.002 | 0.188 | 0.006 | 1 |
|  | Ac_2 vs Ac_4 | 0.010 | 0.898 | 0.032 | 1 |
|  | Ac_2 vs Ac_5 | 0.009 | 0.906 | 0.030 | 1 |
|  | Ac_3 vs Ac_4 | 0.009 | 0.889 | 0.033 | 1 |
|  | Ac_3 vs Ac_5 | 0.005 | 0.592 | 0.021 | 1 |
|  | Ac_4 vs Ac_5 | 0.017 | 2.370 | 0.084 | 1 |

**Table S16. Significant differences in pairwise population genetic differentiation** (estimated from pairwise individual genetic distances through ngsDist) of the different species. *Ad. adscendens*: F 6.79, R² 0.31, df = 5, p < 0.01, *M. maxima*: F 6.85, R² 0.37, df = 4, p < 0.01, *M. phlomoides*: F 3.99, R² 0.19, df = 5, p < 0.01, *M. sanguinea*: F 5.08, R² 0.28, df = 5, p < 0.01, *M. tomentosa*: F 5.02, R² 0.30, df = 4, p < 0.01, *A. costaricensis* F 2.71, R² 0.13, df = 4, p < 0.01. Locality pairs are indicated, as well as Sums of squares, F-values, R², and adjusted p-values (multiple comparisons); significant pairwise differences are highlighted in bold.

| **species** | **pairs** | **SumsOfSqs** | **F** | **R²** | **adj. p-value** |
| --- | --- | --- | --- | --- | --- |
| ***Ad. adscendens*** | **Aa1 vs Aa2** | **0.070** | **4.190** | **0.144** | **0.030** |
|  | **Aa1 vs Aa3** | **0.072** | **4.533** | **0.178** | **0.015** |
|  | **Aa1 vs Aa4** | **0.172** | **9.686** | **0.288** | **0.015** |
|  | **Aa1 vs Aa5** | **0.248** | **14.124** | **0.414** | **0.015** |
|  | **Aa1 vs Aa6** | **0.135** | **9.734** | **0.317** | **0.015** |
|  | Aa2 vs Aa3 | 0.039 | 1.846 | 0.062 | 0.705 |
|  | **Aa2 vs Aa4** | **0.184** | **8.411** | **0.213** | **0.015** |
|  | **Aa2 vs Aa5** | **0.187** | **8.399** | **0.237** | **0.015** |
|  | **Aa2 vs Aa6** | **0.196** | **10.111** | **0.265** | **0.015** |
|  | **Aa3 vs Aa4** | **0.150** | **6.835** | **0.202** | **0.015** |
|  | **Aa3 vs Aa5** | **0.164** | **7.279** | **0.240** | **0.015** |
|  | **Aa3 vs Aa6** | **0.170** | **8.872** | **0.270** | **0.015** |
|  | **Aa4 vs Aa5** | **0.069** | **2.917** | **0.101** | **0.030** |
|  | Aa4 vs Aa6 | 0.050 | 2.449 | 0.083 | 0.060 |
|  | **Aa5 vs Aa6** | **0.128** | **6.183** | **0.212** | **0.015** |
| ***M. maxima*** | Mm_1 vs Mm_2 | 0.010 | 0.831 | 0.053 | 1.00 |
|  | **Mm_1 vs Mm_3** | **0.091** | **8.205** | **0.291** | **0.01** |
|  | **Mm_1 vs Mm_4** | **0.081** | **8.226** | **0.291** | **0.01** |
|  | **Mm_1 vs Mm_5** | **0.015** | **1.405** | **0.069** | **0.01** |
|  | **Mm_2 vs Mm_3** | **0.074** | **6.806** | **0.286** | **0.01** |
|  | **Mm_2 vs Mm_4** | **0.064** | **6.694** | **0.283** | **0.01** |
|  | Mm_2 vs Mm_5 | 0.013 | 1.226 | 0.071 | 0.77 |
|  | **Mm_3 vs Mm_4** | **0.100** | **10.949** | **0.332** | **0.01** |
|  | **Mm_3 vs Mm_5** | **0.114** | **11.829** | **0.360** | **0.01** |
|  | **Mm_4 vs Mm_5** | **0.096** | **11.289** | **0.350** | **0.01** |
| ***M. phlomoides*** | **Mp1-Mp2** | **0.037** | **3.066** | **0.109** | **0.045** |
|  | **Mp1-Mp3** | **0.052** | **3.203** | **0.096** | **0.045** |
|  | Mp1-Mp4 | 0.025 | 1.896 | 0.068 | 0.405 |
|  | **Mp1-Mp5** | **0.076** | **5.455** | **0.173** | **0.015** |
|  | **Mp1-Mp6** | **0.039** | **2.961** | **0.106** | **0.045** |
|  | **Mp2-Mp3** | **0.057** | **3.650** | **0.105** | **0.015** |
|  | **Mp2-Mp4** | **0.046** | **3.625** | **0.118** | **0.015** |
|  | **Mp2-Mp5** | **0.089** | **6.631** | **0.197** | **0.015** |
|  | **Mp2-Mp6** | **0.041** | **3.285** | **0.112** | **0.015** |
|  | **Mp3-Mp4** | **0.066** | **3.993** | **0.111** | **0.015** |
|  | **Mp3-Mp5** | **0.108** | **6.324** | **0.165** | **0.015** |
|  | Mp3-Mp6 | 0.031 | 1.867 | 0.057 | 0.555 |
|  | **Mp4-Mp5** | **0.062** | **4.306** | **0.133** | **0.015** |
|  | **Mp4-Mp6** | **0.044** | **3.238** | **0.107** | **0.015** |
|  | **Mp5-Mp6** | **0.084** | **5.866** | **0.178** | **0.015** |
| ***M. sanguinea*** | **Ms1 vs Ms2** | **0.019** | **2.053** | **0.085** | **0.015** |
|  | Ms1 vs Ms3 | 0.012 | 1.263 | 0.054 | 0.735 |
|  | **Ms1 vs Ms4** | **0.097** | **12.262** | **0.348** | **0.015** |
|  | **Ms1 vs Ms5** | **0.026** | **3.136** | **0.120** | **0.015** |
|  | **Ms1 vs Ms6** | **0.027** | **3.215** | **0.123** | **0.015** |
|  | **Ms2 vs Ms3** | **0.017** | **1.749** | **0.080** | **0.030** |
|  | **Ms2 vs Ms4** | **0.084** | **10.105** | **0.325** | **0.015** |
|  | **Ms2 vs Ms5** | **0.020** | **2.272** | **0.098** | **0.015** |
|  | **Ms2 vs Ms6** | **0.018** | **2.078** | **0.090** | **0.015** |
|  | **Ms3 vs Ms4** | **0.080** | **9.523** | **0.312** | **0.015** |
|  | **Ms3 vs Ms5** | **0.024** | **2.681** | **0.113** | **0.015** |
|  | **Ms3 vs Ms6** | **0.025** | **2.765** | **0.116** | **0.015** |
|  | **Ms4 vs Ms5** | **0.090** | **12.249** | **0.358** | **0.015** |
|  | **Ms4 vs Ms6** | **0.087** | **11.591** | **0.345** | **0.015** |
|  | **Ms5 vs Ms6** | **0.019** | **2.392** | **0.098** | **0.015** |
| ***M. tomentosa*** | **Mt1 vs Mt2** | **0.083** | **5.338** | **0.239** | **0.01** |
|  | **Mt1 vs Mt3** | **0.089** | **5.639** | **0.249** | **0.01** |
|  | **Mt1 vs Mt4** | **0.027** | **1.669** | **0.081** | **0.01** |
|  | **Mt1 vs Mt5** | **0.090** | **6.506** | **0.265** | **0.01** |
|  | **Mt2 vs Mt3** | **0.024** | **1.574** | **0.080** | **0.01** |
|  | **Mt2 vs Mt4** | **0.112** | **7.235** | **0.266** | **0.01** |
|  | **Mt2 vs Mt5** | **0.032** | **2.409** | **0.113** | **0.01** |
|  | **Mt3 vs Mt4** | **0.116** | **7.439** | **0.271** | **0.01** |
|  | **Mt3 vs Mt5** | **0.036** | **2.691** | **0.124** | **0.01** |
|  | **Mt4 vs Mt5** | **0.126** | **9.012** | **0.300** | **0.01** |
| ***A. costaricensis*** | Ac_1 vs Ac_2 | 0.033 | 2.093 | 0.060 | 0.39 |
|  | Ac_1 vs Ac_3 | 0.032 | 2.101 | 0.062 | 0.37 |
|  | **Ac_1 vs Ac_4** | **0.044** | **3.148** | **0.095** | **0.03** |
|  | **Ac_1 vs Ac_5** | **0.041** | **2.698** | **0.078** | **0.01** |
|  | Ac_2 vs Ac_3 | 0.027 | 1.881 | 0.061 | 0.49 |
|  | **Ac_2 vs Ac_4** | **0.038** | **2.987** | **0.100** | **0.01** |
|  | **Ac_2 vs Ac_5** | **0.039** | **2.801** | **0.088** | **0.01** |
|  | **Ac_3 vs Ac_4** | **0.043** | **3.450** | **0.117** | **0.01** |
|  | Ac_3 vs Ac_5 | 0.028 | 2.064 | 0.069 | 0.10 |
|  | **Ac_4 vs Ac_5** | **0.058** | **4.778** | **0.155** | **0.01** |

**Table S17. Pairwise comparison of differences in disparity (genetic diversity**, calculated from covariance matrix on genotype likelihoods) between localities of each species; significant results are highlighted in bold.

| **Species** | **Locality** | **diff** | **lwr** | **upr** | **p adj** |
| --- | --- | --- | --- | --- | --- |
| ***Ad. adscendens*** | **Aa2-Aa1** | **0.051** | **0.027** | **0.076** | **0.000** |
|  | **Aa3-Aa1** | **0.051** | **0.025** | **0.076** | **0.000** |
|  | **Aa4-Aa1** | **0.058** | **0.034** | **0.083** | **0.000** |
|  | **Aa5-Aa1** | **0.062** | **0.036** | **0.088** | **0.000** |
|  | **Aa6-Aa1** | **0.036** | **0.011** | **0.062** | **0.001** |
|  | Aa3-Aa2 | -0.001 | -0.023 | 0.022 | 1.000 |
|  | Aa4-Aa2 | 0.007 | -0.014 | 0.028 | 0.938 |
|  | Aa5-Aa2 | 0.010 | -0.013 | 0.033 | 0.768 |
|  | Aa6-Aa2 | -0.015 | -0.038 | 0.007 | 0.363 |
|  | Aa4-Aa3 | 0.007 | -0.015 | 0.030 | 0.932 |
|  | Aa5-Aa3 | 0.011 | -0.013 | 0.035 | 0.770 |
|  | Aa6-Aa3 | -0.015 | -0.038 | 0.009 | 0.482 |
|  | Aa5-Aa4 | 0.004 | -0.020 | 0.027 | 0.997 |
|  | Aa6-Aa4 | -0.022 | -0.045 | 0.001 | 0.065 |
|  | **Aa6-Aa5** | -0.026 | -0.050 | -0.001 | 0.034 |
| *M. maxima* | Mm_2-Mm_1 | -0.034 | -0.279 | 0.211 | 0.995 |
|  | Mm_3-Mm_1 | -0.055 | -0.268 | 0.158 | 0.948 |
|  | **Mm_4-Mm_1** | **-0.216** | **-0.429** | **-0.003** | **0.045** |
|  | Mm_5-Mm_1 | -0.161 | -0.378 | 0.056 | 0.236 |
|  | Mm_3-Mm_2 | -0.021 | -0.257 | 0.215 | 0.999 |
|  | Mm_4-Mm_2 | -0.182 | -0.419 | 0.054 | 0.202 |
|  | Mm_5-Mm_2 | -0.127 | -0.368 | 0.113 | 0.567 |
|  | Mm_4-Mm_3 | -0.161 | -0.364 | 0.042 | 0.178 |
|  | Mm_5-Mm_3 | -0.106 | -0.314 | 0.101 | 0.598 |
|  | Mm_5-Mm_4 | 0.055 | -0.152 | 0.263 | 0.943 |
| *M. phlomoides* | Mp1-Mp2 | -0.005 | -0.036 | 0.026 | 0.998 |
|  | **Mp1-Mp3** | **0.030** | **0.001** | **0.059** | **0.042** |
|  | Mp1-Mp4 | 0.007 | -0.024 | 0.037 | 0.986 |
|  | Mp1-Mp5 | 0.015 | -0.015 | 0.046 | 0.688 |
|  | Mp1-Mp6 | 0.006 | -0.025 | 0.037 | 0.992 |
|  | **Mp2-Mp3** | **0.034** | **0.006** | **0.063** | **0.008** |
|  | Mp2-Mp4 | 0.012 | -0.018 | 0.041 | 0.864 |
|  | Mp2-Mp5 | 0.020 | -0.010 | 0.050 | 0.377 |
|  | Mp2-Mp6 | 0.011 | -0.019 | 0.041 | 0.900 |
|  | Mp3-Mp4 | -0.023 | -0.050 | 0.005 | 0.174 |
|  | Mp3-Mp5 | -0.014 | -0.042 | 0.013 | 0.662 |
|  | Mp3-Mp6 | -0.023 | -0.052 | 0.005 | 0.163 |
|  | Mp4-Mp5 | 0.008 | -0.021 | 0.038 | 0.961 |
|  | Mp4-Mp6 | -0.001 | -0.031 | 0.029 | 1.000 |
|  | Mp5-Mp6 | -0.009 | -0.039 | 0.021 | 0.948 |
| *M. sanguinea* | Ms2-Ms1 | 0.005 | -0.015 | 0.025 | 0.977 |
|  | Ms3-Ms1 | 0.005 | -0.015 | 0.025 | 0.978 |
|  | Ms4-Ms1 | -0.010 | -0.029 | 0.010 | 0.663 |
|  | Ms5-Ms1 | -0.005 | -0.025 | 0.014 | 0.965 |
|  | Ms6-Ms1 | -0.005 | -0.025 | 0.014 | 0.969 |
|  | Ms3-Ms2 | 0.000 | -0.021 | 0.021 | 1 |
|  | Ms4-Ms2 | -0.015 | -0.035 | 0.005 | 0.271 |
|  | Ms5-Ms2 | -0.010 | -0.031 | 0.010 | 0.670 |
|  | Ms6-Ms2 | -0.010 | -0.031 | 0.010 | 0.683 |
|  | Ms4-Ms3 | -0.015 | -0.035 | 0.005 | 0.272 |
|  | Ms5-Ms3 | -0.010 | -0.031 | 0.010 | 0.672 |
|  | Ms6-Ms3 | -0.010 | -0.031 | 0.010 | 0.684 |
|  | Ms5-Ms4 | 0.005 | -0.015 | 0.024 | 0.983 |
|  | Ms6-Ms4 | 0.005 | -0.015 | 0.025 | 0.981 |
|  | Ms6-Ms5 | 0.000 | -0.020 | 0.020 | 1.000 |
| ***M. tomentosa*** | **Mt2-Mt1** | **-0.090** | **-0.179** | **0.000** | **0.049** |
|  | Mt3-Mt1 | -0.082 | -0.172 | 0.007 | 0.084 |
|  | Mt4-Mt1 | -0.015 | -0.101 | 0.071 | 0.989 |
|  | **Mt5-Mt1** | **-0.178** | **-0.265** | **-0.090** | **0.000** |
|  | Mt3-Mt2 | 0.007 | -0.080 | 0.095 | 0.999 |
|  | Mt4-Mt2 | 0.075 | -0.008 | 0.159 | 0.096 |
|  | **Mt5-Mt2** | **-0.088** | **-0.173** | **-0.003** | **0.041** |
|  | Mt4-Mt3 | 0.068 | -0.016 | 0.151 | 0.161 |
|  | **Mt5-Mt3** | **-0.095** | **-0.180** | **-0.010** | **0.022** |
|  | **Mt5-Mt4** | **-0.163** | **-0.244** | **-0.082** | **0.000** |
| *A. costaricensis* | Ac_2-Ac_1 | -0.005 | -0.040 | 0.029 | 0.993 |
|  | Ac_3-Ac_1 | -0.006 | -0.042 | 0.029 | 0.986 |
|  | Ac_4-Ac_1 | -0.021 | -0.058 | 0.016 | 0.498 |
|  | Ac_5-Ac_1 | -0.006 | -0.041 | 0.029 | 0.990 |
|  | Ac_3-Ac_2 | -0.001 | -0.038 | 0.035 | 1.000 |
|  | Ac_4-Ac_2 | -0.016 | -0.054 | 0.022 | 0.774 |
|  | Ac_5-Ac_2 | -0.001 | -0.037 | 0.036 | 1.000 |
|  | Ac_4-Ac_3 | -0.015 | -0.053 | 0.024 | 0.825 |
|  | Ac_5-Ac_3 | 0.001 | -0.037 | 0.038 | 1.000 |
|  | Ac_5-Ac_4 | 0.015 | -0.023 | 0.054 | 0.805 |

**Table S18. Pairwise comparison of differences in disparity (genetic diversity**, calculated from individual genetic distances through ngsDist) between localities of each species; significant results are highlighted in bold.

| **Species** | **Localities** | **diff** | **lwr** | **upr** | **p adj** |
| --- | --- | --- | --- | --- | --- |
| ***Ad. adscendens*** | **Aa2-Aa1** | **0.051** | **0.027** | **0.076** | **0.000** |
|  | **Aa3-Aa1** | **0.051** | **0.025** | **0.076** | **0.000** |
|  | **Aa4-Aa1** | **0.058** | **0.034** | **0.083** | **0.000** |
|  | **Aa5-Aa1** | **0.062** | **0.036** | **0.088** | **0.000** |
|  | **Aa6-Aa1** | **0.036** | **0.011** | **0.062** | **0.001** |
|  | Aa3-Aa2 | -0.001 | -0.023 | 0.022 | 1.000 |
|  | Aa4-Aa2 | 0.007 | -0.014 | 0.028 | 0.938 |
|  | Aa5-Aa2 | 0.010 | -0.013 | 0.033 | 0.768 |
|  | Aa6-Aa2 | -0.015 | -0.038 | 0.007 | 0.363 |
|  | Aa4-Aa3 | 0.007 | -0.015 | 0.030 | 0.932 |
|  | Aa5-Aa3 | 0.011 | -0.013 | 0.035 | 0.770 |
|  | Aa6-Aa3 | -0.015 | -0.038 | 0.009 | 0.482 |
|  | Aa5-Aa4 | 0.004 | -0.020 | 0.027 | 0.997 |
|  | Aa6-Aa4 | -0.022 | -0.045 | 0.001 | 0.065 |
|  | **Aa6-Aa5** | **-0.026** | **-0.050** | **-0.001** | **0.034** |
| ***M. maxima*** | Mm2-Mm1 | -0.005 | -0.028 | 0.019 | 0.980 |
|  | Mm3-Mm1 | -0.009 | -0.029 | 0.011 | 0.721 |
|  | Mm4-Mm1 | -0.019 | -0.040 | 0.001 | 0.067 |
|  | Mm5-Mm1 | -0.015 | -0.035 | 0.006 | 0.283 |
|  | Mm3-Mm2 | -0.004 | -0.027 | 0.018 | 0.982 |
|  | Mm4-Mm2 | -0.015 | -0.037 | 0.008 | 0.353 |
|  | Mm5-Mm2 | -0.010 | -0.033 | 0.013 | 0.733 |
|  | Mm4-Mm3 | -0.010 | -0.030 | 0.009 | 0.547 |
|  | Mm5-Mm3 | -0.006 | -0.025 | 0.014 | 0.927 |
|  | Mm5-Mm4 | 0.005 | -0.015 | 0.025 | 0.957 |
| ***M. phlomoides*** | Mp1-Mp2 | -0.050 | -0.343 | 0.243 | 0.996 |
|  | **Mp1-Mp3** | **0.291** | **0.017** | **0.565** | **0.031** |
|  | Mp1-Mp4 | 0.058 | -0.230 | 0.346 | 0.991 |
|  | Mp1-Mp5 | 0.123 | -0.165 | 0.411 | 0.815 |
|  | Mp1-Mp6 | 0.086 | -0.207 | 0.379 | 0.956 |
|  | **Mp2-Mp3** | **0.341** | **0.073** | **0.609** | **0.005** |
|  | Mp2-Mp4 | 0.109 | -0.174 | 0.391 | 0.872 |
|  | Mp2-Mp5 | 0.173 | -0.110 | 0.456 | 0.480 |
|  | Mp2-Mp6 | 0.136 | -0.151 | 0.424 | 0.738 |
|  | Mp3-Mp4 | -0.233 | -0.495 | 0.030 | 0.113 |
|  | Mp3-Mp5 | -0.168 | -0.431 | 0.095 | 0.430 |
|  | Mp3-Mp6 | -0.205 | -0.473 | 0.063 | 0.234 |
|  | Mp4-Mp5 | 0.065 | -0.213 | 0.342 | 0.984 |
|  | Mp4-Mp6 | 0.028 | -0.255 | 0.310 | 1.000 |
|  | Mp5-Mp6 | -0.037 | -0.320 | 0.246 | 0.999 |
| ***M. sanguinea*** | Ms2-Ms1 | 0.016 | -0.307 | 0.339 | 1.000 |
|  | Ms3-Ms1 | 0.090 | -0.233 | 0.413 | 0.964 |
|  | Ms4-Ms1 | -0.170 | -0.486 | 0.145 | 0.611 |
|  | Ms5-Ms1 | -0.097 | -0.413 | 0.218 | 0.944 |
|  | Ms6-Ms1 | -0.097 | -0.413 | 0.219 | 0.944 |
|  | Ms3-Ms2 | 0.074 | -0.262 | 0.410 | 0.987 |
|  | Ms4-Ms2 | -0.186 | -0.515 | 0.143 | 0.563 |
|  | Ms5-Ms2 | -0.113 | -0.442 | 0.216 | 0.914 |
|  | Ms6-Ms2 | -0.113 | -0.442 | 0.216 | 0.914 |
|  | Ms4-Ms3 | -0.260 | -0.589 | 0.069 | 0.201 |
|  | Ms5-Ms3 | -0.187 | -0.516 | 0.142 | 0.557 |
|  | Ms6-Ms3 | -0.187 | -0.516 | 0.142 | 0.559 |
|  | Ms5-Ms4 | 0.073 | -0.249 | 0.395 | 0.985 |
|  | Ms6-Ms4 | 0.073 | -0.249 | 0.395 | 0.985 |
|  | Ms6-Ms5 | 0.000 | -0.322 | 0.322 | 1 |
| ***M. tomentosa*** | Mt2-Mt1 | -0.005 | -0.016 | 0.005 | 0.599 |
|  | Mt3-Mt1 | -0.004 | -0.014 | 0.006 | 0.772 |
|  | Mt4-Mt1 | 0.000 | -0.009 | 0.010 | 1.000 |
|  | **Mt5-Mt1** | **-0.018** | **-0.028** | **-0.008** | **0.000** |
|  | Mt3-Mt2 | 0.001 | -0.009 | 0.011 | 0.998 |
|  | Mt4-Mt2 | 0.006 | -0.004 | 0.015 | 0.444 |
|  | **Mt5-Mt2** | **-0.013** | **-0.023** | **-0.003** | **0.004** |
|  | Mt4-Mt3 | 0.005 | -0.005 | 0.014 | 0.636 |
|  | **Mt5-Mt3** | **-0.014** | **-0.024** | **-0.004** | **0.001** |
|  | **Mt5-Mt4** | **-0.019** | **-0.028** | **-0.009** | **0.000** |
| **A. costaricensis** | Ac2-Ac1 | -0.071 | -0.451 | 0.308 | 0.984 |
|  | Ac3-Ac1 | -0.095 | -0.481 | 0.291 | 0.958 |
|  | Ac4-Ac1 | -0.265 | -0.667 | 0.137 | 0.357 |
|  | Ac5-Ac1 | -0.133 | -0.519 | 0.253 | 0.871 |
|  | Ac3-Ac2 | -0.024 | -0.425 | 0.378 | 1.000 |
|  | Ac4-Ac2 | -0.194 | -0.611 | 0.224 | 0.693 |
|  | Ac5-Ac2 | -0.062 | -0.463 | 0.340 | 0.993 |
|  | Ac4-Ac3 | -0.170 | -0.593 | 0.253 | 0.794 |
|  | Ac5-Ac3 | -0.038 | -0.446 | 0.370 | 0.999 |
|  | Ac5-Ac4 | 0.132 | -0.291 | 0.556 | 0.906 |

**Table S19. Results of Mantel’s tests on the impact of IBD, IBR_Terrain_, IBR_Habitat_, IBI and IBE on normalized population genetic differentiation (F_ST_).** Significant isolation by distance and/or resistance in all species but *A. costaricensis*, no IBE, the highest significant R² for each species is highlighted in bolt, significant values in italics.

| **species** | **IBD** | | **IBRTerrain** | | **IBRHabitat** | | **IBI** | | **IBE** | |
| --- | --- | --- | --- | --- | --- | --- | --- | --- | --- | --- |
|  | **R²** | ***p*** | **R²** | ***p*** | **R²** | ***p*** | **R²** | ***p*** | **R²** | ***p*** |
| ***Ad. adscendens*** | ***0.785*** | ***0.047*** | 0.821 | 0.053 | 0.017 | 0.470 | *0.796* | *0.048* | 0.556 | 0.055 |
| ***M. maxima*** | *0.687* | *0.017* | *0.673* | *0.017* | ***0.777*** | ***0.023*** | *0.729* | *0.016* | 0.091 | 0.394 |
| ***M. phlomoides*** | ***0.815*** | ***0.006*** | *0.784* | *0.008* | 0.363 | 0.171 | *0.808* | *0.002* | -0.108 | 0.622 |
| ***M. sanguinea*** | ***0.987*** | ***0.005*** | *0.987* | *0.007* | *0.984* | *0.01* | *0.985* | *0.011* | -0.150 | 0.348 |
| ***M. tomentosa*** | **0.951** | **0.015** | *0.948* | *0.016* | *0.941* | *0.024* | *0.950* | *0.017* | -0.016 | 0.434 |
| ***A. costaricensis*** | **0.750** | **0.055** | 0.675 | 0.074 | -0.002 | 0.547 | 0.417 | 0.073 | -0.093 | 0.632 |

**Table S20. Results of Multiple Matrix Regression with Randomization** testing for the joint impact of the IBD/IBR matrix with highest R² (>R² from Table 1) and IBE on population genetic differentiation (F_ST)_.

| **species** |  | **Full model** | | | **IBD or IBR** | | **IBE** | |
| --- | --- | --- | --- | --- | --- | --- | --- | --- |
|  | **> R²** | **F-value** | **p-value** | **R²** | **t-value** | **p-value** | **t-value** | **p-value** |
| ***Ad. adscendens*** | **IBI** | 15.24 | 0.079 | 0.717 | 4.164 | 0.075 | 1.895 | 0.065 |
| ***M. maxima*** | **IBRH** | 5.37 | 0.069 | 0.605 | 3.255 | 0.063 | 0.142 | 0.974 |
| ***M. phlomoides*** | **IBD** | 12.419 | 0.003 | 0.674 | 4.941 | *0.003* | 0.600 | 0.507 |
| ***M. sanguinea*** | **IBD** | 252.012 | 0.006 | 0.977 | 22.190 | *0.006* | -0.097 | 0.978 |
| ***M. tomentosa*** | **IBD** | 64.398 | 0.007 | 0.948 | 11.347 | *0.007* | -2.475 | 0.095 |
| ***A. costaricensis*** | **IBD** | 4.806 | 0.121 | 0.579 | 3.077 | 0.110 | -0.526 | 0.597 |

**Table S21. Results of Generalized Dissimilarity Modelling**, testing for the relative effect of IBD, IBR (IBH – IBRHabitat; IBT – IBRTerrain; IBI) and IBE on population genetic differentiation. In the full model, all five explanatory variables are used; next, single variables are removed sequentially to calculate the contribution of each variable to model fit, the explanatory variables retained in each model are given in the header. A large change in model deviance and percent of deviance explained indicates the removal of an explanatory variable with strong effect on *F_ST_*.

| ***Ad. adscendens*** | **fullModel** | **IBD+IBE+IBT+IBI** | **IBD+IBE+IBT** | **IBD+IBE** | **IBD** |
| --- | --- | --- | --- | --- | --- |
| **Model deviance** | 0.469 | 0.469 | 0.469 | 0.474 | 0.525 |
| **Percent deviance explained** | 80.128 | 80.128 | 80.128 | 79.937 | 77.771 |
| **Model p-value** | 0.019 | 0.012 | 0.007 | 0.018 | 0.028 |
| **Fitted permutations** | 425 | 433 | 422 | 433 | 286 |
|  |  |  |  |  |  |
| ***M. maxima*** | **fullModel** | **IBD+IBH+IBE+IBI** | **IBD+IBH+IBE** | **IBD+IBH** | **IBH** |
| **Model deviance** | 0.178 | 0.178 | 0.178 | 0.182 | 0.186 |
| **Percent deviance explained** | 87.952 | 87.952 | 87.952 | 87.678 | 87.438 |
| **Model p-value** | 0 | 0 | 0 | 0 | 0 |
| **Fitted permutations** | 370 | 375 | 369 | 362 | 188 |
|  |  |  |  |  |  |
| ***M. phlomoides*** | **fullModel** | **IBD+IBE+IBI+IBT** | **IBD+IBE+IBI** | **IBD+IBE** | **IBD** |
| **Model deviance** | 0.132 | 0.132 | 0.132 | 0.132 | 0.497 |
| **Percent deviance explained** | 73.537 | 73.537 | 73.537 | 73.534 | 0.060 |
| **Model p-value** | 0.018 | 0.003 | 0.021 | 0.013 | 1 |
| **Fitted permutations** | 389 | 356 | 380 | 381 | 157 |
|  |  |  |  |  |  |
| ***M. sanguinea*** | **fullModel** | **IBD+IBE+IBH+IBT** | **IBD+IBE+IBH** | **IBD+IBE** | **IBD** |
| **Model deviance** | 0.035 | 0.035 | 0.035 | 0.035 | 0.059 |
| **Percent deviance explained** | 98.702 | 98.702 | 98.702 | 98.702 | 97.835 |
| **Model p-value** | 0.004 | 0.025 | 0.013 | 0 | 0.020 |
| **Fitted permutations** | 256 | 236 | 225 | 249 | 147 |
|  |  |  |  |  |  |
| ***M. tomentosa*** | **fullModel** | **IBD+IBI+IBH+IBE** | **IBD+IBI+IBH** | **IBD+IBI** | **IBI** |
| **Model deviance** | 0.023 | 0.023 | 0.023 | 0.023 | 0.038 |
| **Percent deviance explained** | 96.569 | 96.569 | 96.569 | 96.569 | 94.451 |
| **Model p-value** | 0 | 0 | 0 | 0 | 0 |
| **Fitted permutations** | 350 | 328 | 362 | 354 | 299 |
|  |  |  |  |  |  |
| ***A. costaricensis*** | **fullModel** | **IBD+IBE+IBI+IBH** | **IBD+IBE+IBI** | **IBD+IBE** | **IBD** |
| **Model deviance** | 0.080 | 0.080 | 0.080 | 0.080 | 0.080 |
| **Percent deviance explained** | 64.663 | 64.663 | 64.663 | 64.663 | 64.663 |
| **Model p-value** | 0.291 | 0.295 | 0.294 | 0.330 | 0.111 |
| **Fitted permutations** | 437 | 437 | 445 | 437 | 316 |


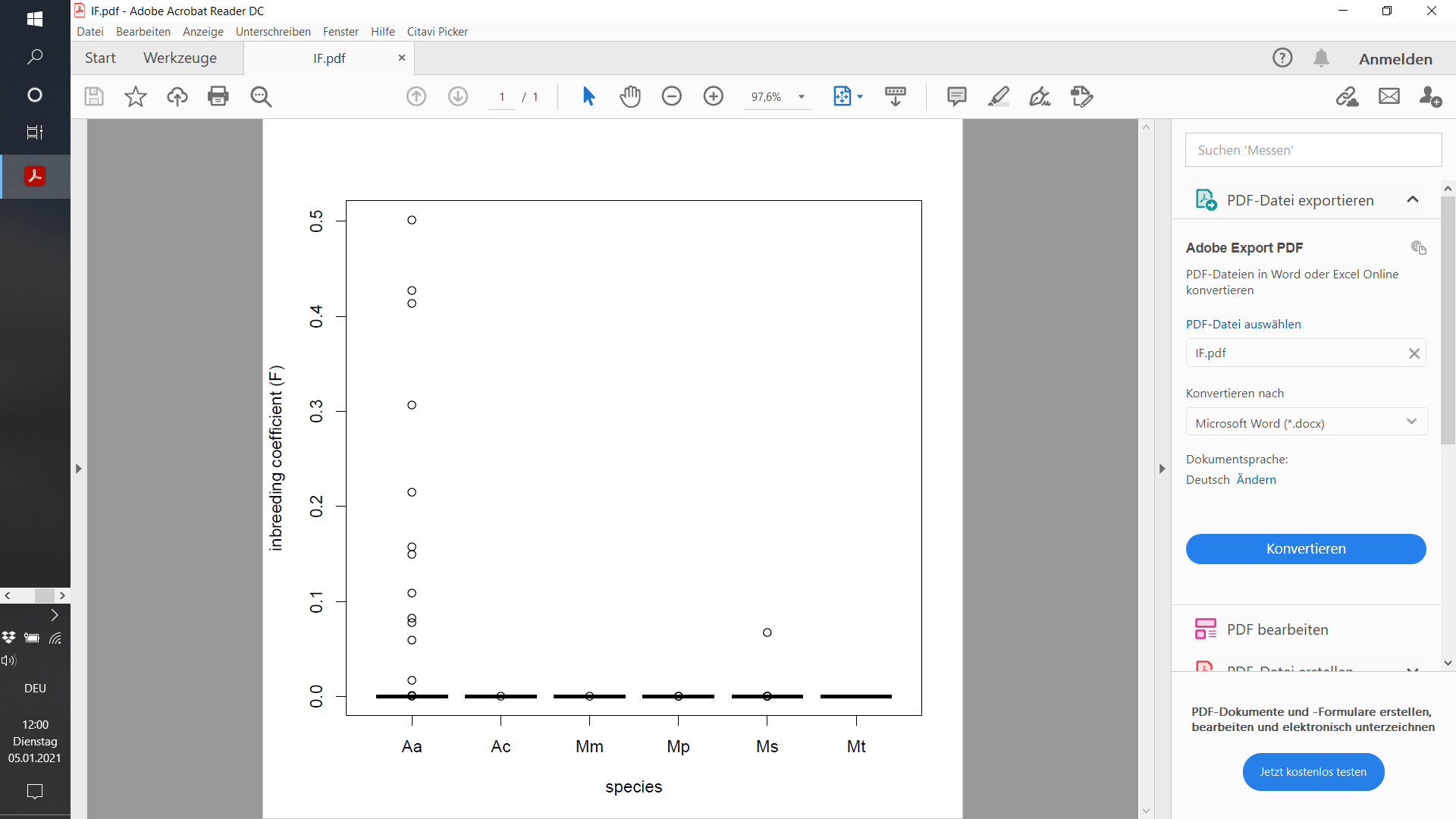


**Figure S4. Per-individual inbreeding coefficient (F) for the six study species**; *Ad. adscendens* (Aa) showed intermediate levels of inbreeding (> 0.05 – 0.51) in eleven individuals, which mostly belonged to localities 1 and 2. Inbreeding was very low in all other localities.


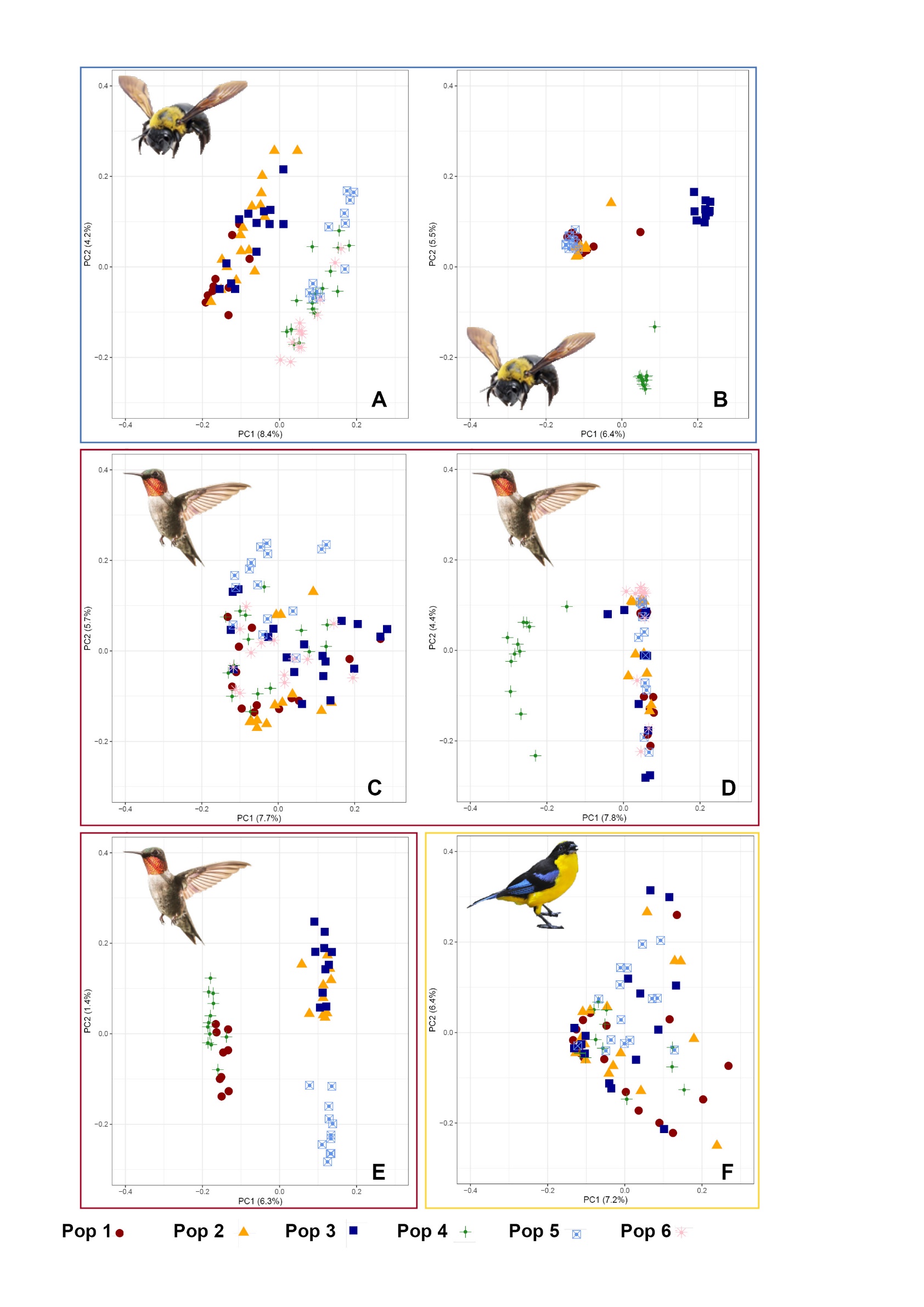


**Figure S5. Population genetic differentiation of the six study species.** The total genetic variation explained by PCA-axes 1 and 2 was: *Ad. adscendens* 15.9%, *M. maxima* 11.6%, *M. phlomoides* 14.4%, *M. sanguinea* 11.4%, *M. tomentosa* 7.1%, *A. costaricensis* 12.9%. (A) Bee-pollinated *Adelobotrys adscendens* with two clusters, localities 1, 2 and 3 in Northern and Central Costa Rica and localities 4, 5 and 6 in Southern Costa Rica; locality 1 being significantly less disparate than the others. (B) Bee-pollinated *Meriania maxima* with three clusters: localities 3 and 4 (only 20 km apart) differing significantly from each other and all other localities, and one cluster comprising three intermixed, undifferentiated localities (1, 2 and 5 from North-Central Ecuador). (C) In hummingbird-bat-pollinated *M. phlomoides*, clustering was weak, localities 1, 2, 3, 4 and 6 are intermixed on PC1 and PC2 and only locality 5 differed significantly from 1, 2, 4 and 6; locality 3 is significantly more variable than the others. (D) In hummingbird-rodent-pollinated *M. sanguinea*, five localities from Southern Ecuador clustered together and were significantly different from locality 4 (Northern Ecuador). (E) Hummingbird-bat-pollinated *M. tomentosa* with three clusters, localities 1 and 4 (Norther Ecuador) differ significantly from localities 2 and 3 (Southern Ecuador) and locality 5 (Southern Ecuador). (F) All localities were intermixed on PC1 and PC2 in passerine-pollinated *A. costaricensis* and did not differ significantly.


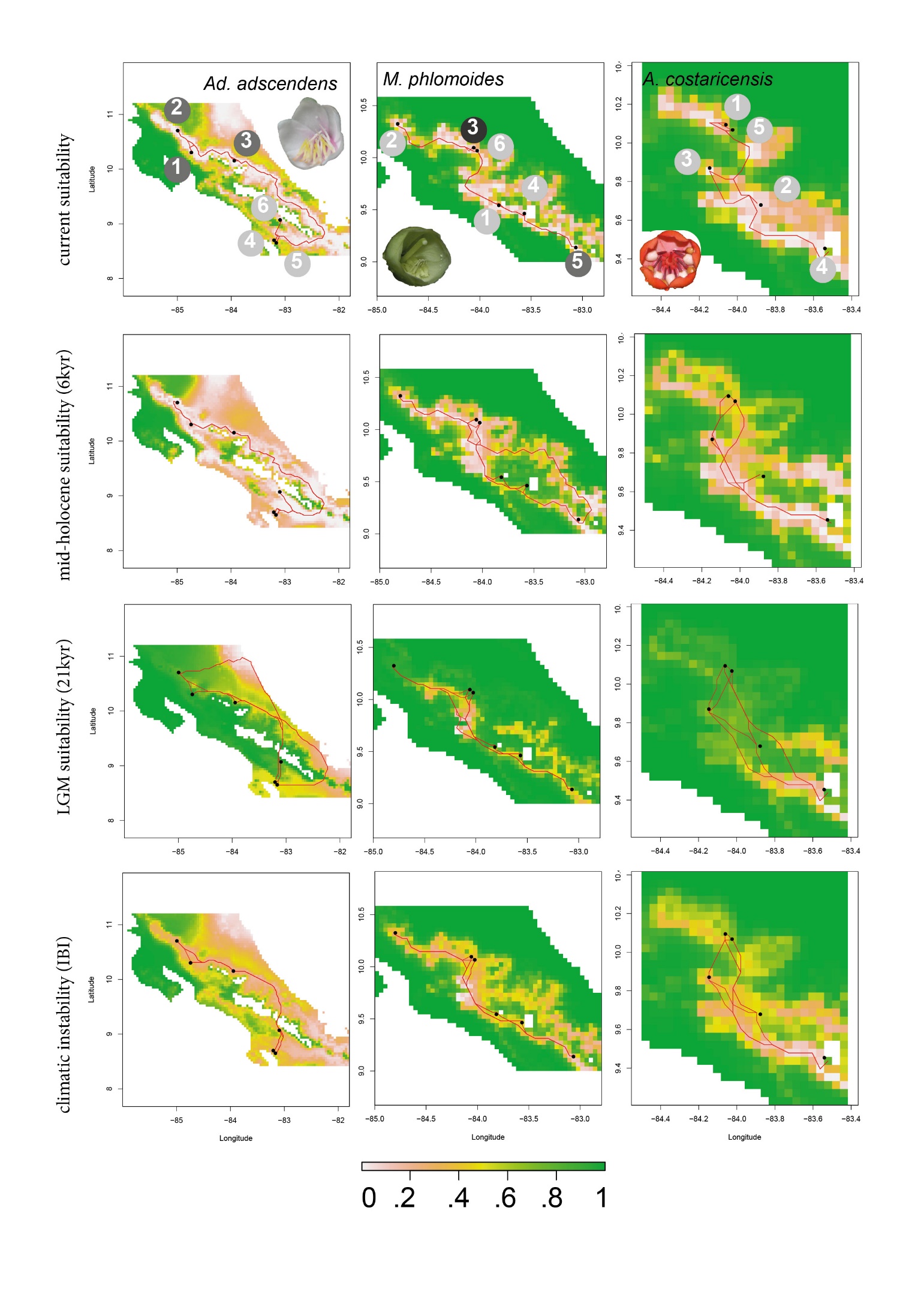


**Figure S6. Habitat resistance and least-cost paths for current climate, mid-Holocene (6 k years), the LGM (21 k years) and climatic instability (IBI) for the study localities of the Costa Rican study species**, 0 indicates low habitat resistance (i.e. highly suitable habitat), 1 indicates high habitat resistance (i.e. unsuitable habitat).


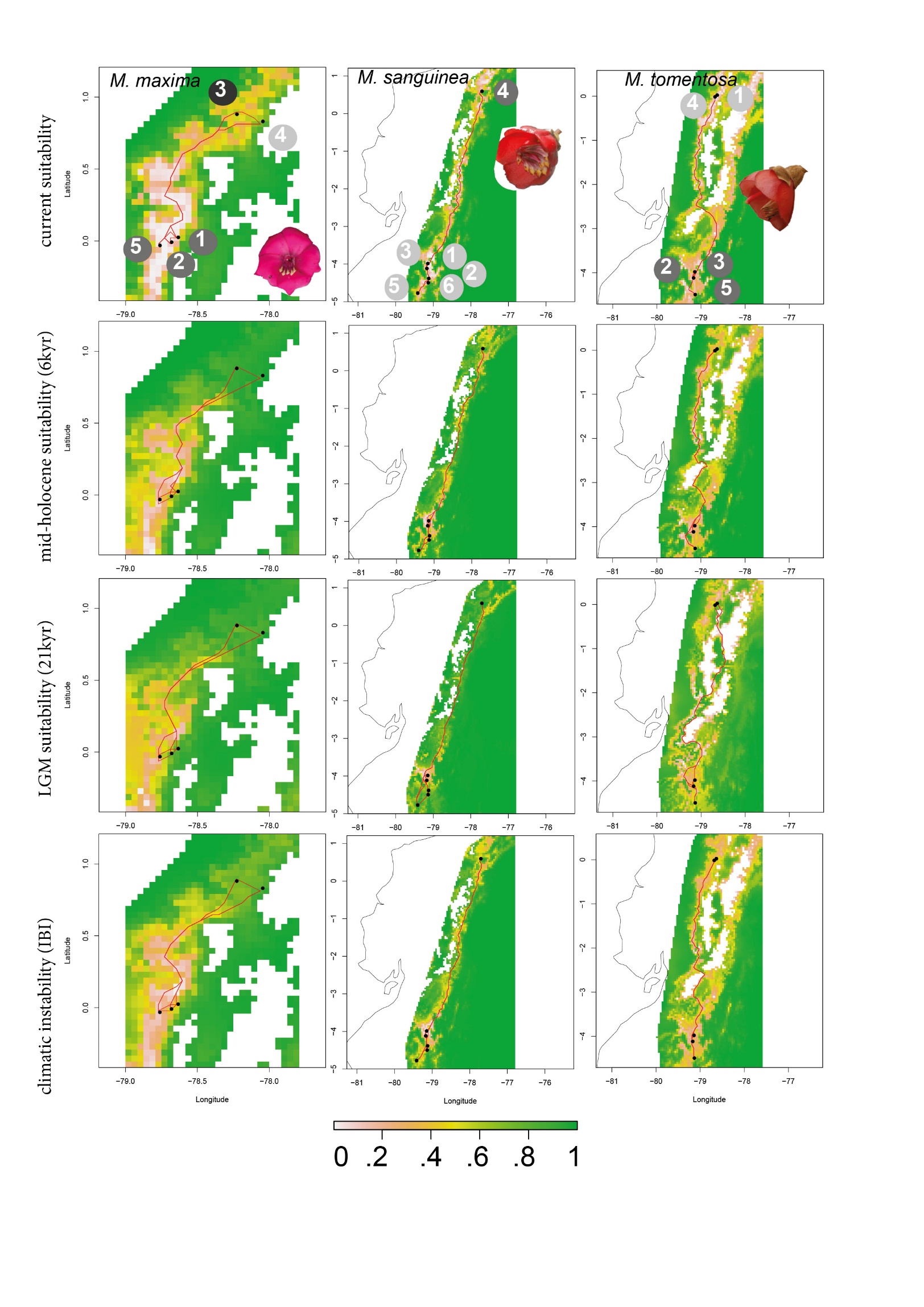


**Figure S7. Habitat resistance and least-cost paths for current climate, mid-Holocene (6 k years), the LGM (21 k years) and climatic instability (IBI) for the study localities of the Ecuadorian study species**, 0 indicates low habitat resistance (i.e. highly suitable habitat), 1 indicates high habitat resistance (i.e. unsuitable habitat).

**Figure S8.** **GDM-fitted I splines** for the five explanatory variables IBD, IBRTerrain (abbreviated as IBRt), IBRHabitat (abbreviated as IBRc), IBI and IBE for bee-pollinated *Ad. adscendens*.

**Figure S9.** **GDM-fitted I splines** for the five explanatory variables IBD, IBRTerrain (abbreviated as IBRt), IBRHabitat (abbreviated as IBRc), IBI and IBE for bee-pollinated *M. maxima*.

**Figure S10.** **GDM-fitted I splines** for the five explanatory variables IBD, IBRTerrain (abbreviated as IBRt), IBRHabitat (abbreviated as IBRc), IBI and IBE for hummingbird-bat-pollinated *M. phlomoides*.

**Figure S11.** **GDM-fitted I splines** for the five explanatory variables IBD, IBRTerrain (abbreviated as IBRt), IBRHabitat (abbreviated as IBRc), IBI and IBE for hummingbird-rodent-bat pollinated *M. sanguinea*.

**Figure S12.** **GDM-fitted I splines** for the five explanatory variables IBD, IBRTerrain (abbreviated as IBRt), IBRHabitat (abbreviated as IBRc), IBI and IBE for hummingbird-bat-pollinated *M. tomentosa*.

**Figure S13.** **GDM-fitted I splines** for the five explanatory variables IBD, IBRTerrain (abbreviated as IBRt), IBRHabitat (abbreviated as IBRc), IBI and IBE for passerine-pollinated *A. costaricensis*; none having a significant effect on population genetic differentiation.


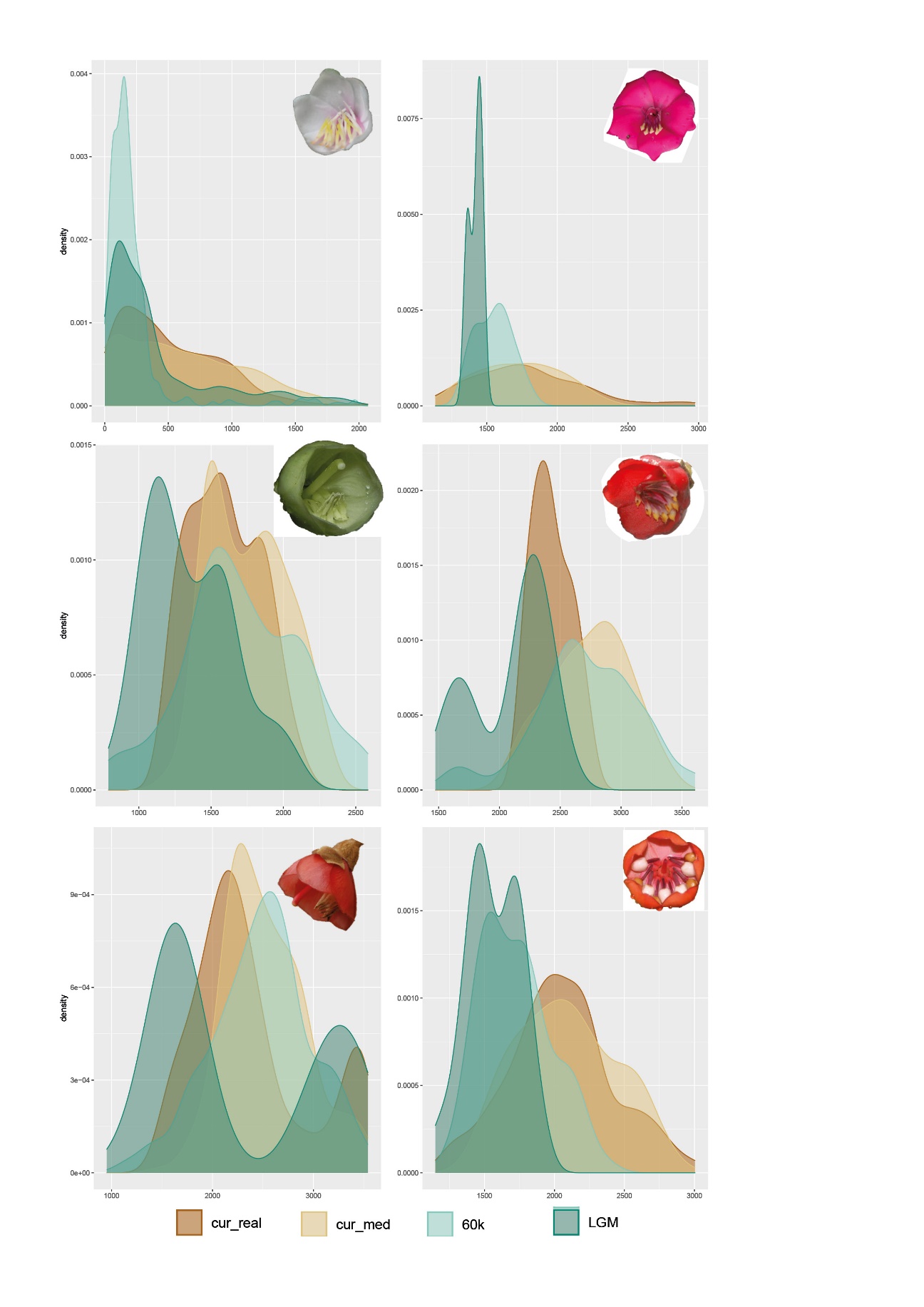


**Figure S14. Changes in elevational distribution of the six study species from LGM, mid-Holocene to today**. Cur_real – current elevational distribution of actual GBIF occurrences (see Table S5 for number of occurrences of each species). Cur_med – current elevational distribution of a random sample of cells above median habitat suitability per species (number of random samples corresponds to number of occurrences per species, Table S5). 60k – mid-Holocene elevational distribution of random sample of cells above median habitat suitability per species (note that fewer cells above median current suitability were available for the following species: *M. maxima* – 13, *M. sanguinea* – 35). LGM – LGM elevational distribution of random sample of cells above median habitat suitability per species (note that fewer cells above median current suitability were available for the following species: *M. maxima* – 3, *M. sanguinea* – 54, *A. costaricensis* - 38).
